# Expression of Cancer Testis Antigens in Tumor-Adjacent Normal Liver Is Associated with Post-Resection Recurrence of Hepatocellular Carcinoma

**DOI:** 10.3390/cancers13102499

**Published:** 2021-05-20

**Authors:** Lisanne Noordam, Zhouhong Ge, Hadiye Özturk, Michail Doukas, Shanta Mancham, Patrick P. C. Boor, Lucia Campos Carrascosa, Guoying Zhou, Thierry P. P. van den Bosch, Qiuwei Pan, Jan N. M. IJzermans, Marco J. Bruno, Dave Sprengers, Jaap Kwekkeboom

**Affiliations:** 1 Department of Gastroenterology and Hepatology, Erasmus MC-University Medical Center, 3000 CA Rotterdam, The Netherlands; l.noordam.1@erasmusmc.nl (L.N.); z.ge@erasmusmc.nl (Z.G.); hozturk@amphia.nl (H.Ö.); s.mancham@erasmusmc.nl (S.M.); p.boor@erasmusmc.nl (P.P.C.B.); lucia.campos_carrascosa@roche.com (L.C.C.); g.zhou@erasmusmc.nl (G.Z.); q.pan@erasmusmc.nl (Q.P.); m.bruno@erasmusmc.nl (M.J.B.); d.sprengers@erasmusmc.nl (D.S.); 2 Department of Pathology, Erasmus MC-University Medical Center, 3000 CA Rotterdam, The Netherlands; m.doukas@erasmusmc.nl (M.D.); t.vandenbosch@erasmusmc.nl (T.P.P.v.d.B.); 3 Department of Surgery, Erasmus MC-University Medical Center, 3000 CA Rotterdam, The Netherlands; J.ijzermans@erasmusmc.nl

**Keywords:** liver neoplasms, cancer testis antigens, prognosis, neoplasm recurrence, immunotherapy

## Abstract

**Simple Summary:**

High recurrence rates after resection of liver cancer (hepatocellular carcinoma) with curative intent impair clinical outcomes of patients diagnosed with liver cancer. Cancer/testis antigens (CTAs) are expressed in cancer and can serve as therapeutic targets. We identified 12 CTAs expressed in 80% of liver cancer patients, and each one individually in at least 10%. Furthermore, we found that patients with expression of CTAs in macroscopically tumor-free liver tissue, experience more tumor recurrence and poor survival after surgical tumor removal. The increased risk of tumor recurrence in patients with CTA expression in tumor-free liver suggests that these patients already have micro-metastasis at the time of operation. These CTA-expressing (pre-)malignant cells may thus be a source of liver cancer recurrence, reflecting the relevance of targeting these to prevent liver cancer recurrence.

**Abstract:**

High recurrence rates after resection of hepatocellular carcinoma (HCC) with curative intent impair clinical outcomes of HCC. Cancer/testis antigens (CTAs) are suitable targets for cancer immunotherapy if selectively expressed in tumor cells. The aims were to identify CTAs that are frequently and selectively expressed in HCC-tumors, and to investigate whether CTAs could serve as biomarkers for occult metastasis. Tumor and paired tumor-free liver (TFL) tissues of HCC-patients and healthy tissues were assessed for mRNA expression of 49 CTAs by RT-qPCR and protein expression of five CTAs by immunohistochemistry. Twelve CTA-mRNAs were expressed in ≥10% of HCC-tumors and not in healthy tissues except testis. In tumors, mRNA and protein of ≥ 1 CTA was expressed in 78% and 71% of HCC-patients, respectively. In TFL, CTA mRNA and protein was found in 45% and 30% of HCC-patients, respectively. Interestingly, CTA-expression in TFL was an independent negative prognostic factor for post-resection HCC-recurrence and survival. We established a panel of 12 testis-restricted CTAs expressed in tumors of most HCC-patients. The increased risk of HCC-recurrence in patients with CTA expression in TFL, suggests that CTA-expressing (pre-)malignant cells may be a source of HCC-recurrence, reflecting the relevance of targeting these to prevent HCC-recurrence.

## 1. Introduction

Liver cancer is the fourth leading cause of cancer related death, with hepatocellular carcinoma (HCC) being the most common subtype [1]. HCC is often diagnosed at advanced stage and these patients can only be offered palliative therapies [2,3]. However, with the help of intensive monitoring, at-risk-patients can be diagnosed at an early stage and can therefore be treated with curative intent: either by surgical resection or radiofrequency ablation. However, recurrence rates are high and currently no therapies are available to prevent recurrence. Patients experiencing early recurrence likely have occult multifocality at the time of resection, whereas late recurrences are more likely to represent de novo tumors [4,5,6]. Several clinicopathological factors, such as tumor size and vascular invasion, have been used to predict clinical outcome after surgery, but none have consequences for the management of HCC after surgical treatment [7]. It remains of great importance to identify occult metastasis at the time of resection to allow identification of patients at risk for recurrence, ideally by targetable tumor markers. Once occult micro-metastasis or de novo (pre-)malignant lesions can be characterized, therapeutic approaches targeting these markers may be developed to prevent tumor recurrence.

Cancer testis antigens (CTAs) are expressed in immune-privileged germ cells and in cancer cells of various histological subtypes [8]. Based on their expression profile in adult healthy tissues, they are classified into testis-restricted, testis/brain-restricted, and testis-selective CTAs with the last group having additional expression in somatic tissues [9]. Since testis-restricted CTAs lack expression in healthy adult tissues, and have the potential to induce antitumor immune responses, they are considered ideal targets for cancer immunotherapy [8,10]. Moreover, as testis-restricted CTAs are not expressed in healthy, tumor-free tissues, sensitive techniques detecting these CTAs can potentially be used to recognize occult metastasis in surrounding macro- and microscopically tumor-free tissue.

The aims of this study were: (1) To establish a panel of CTAs that are frequently and selectively expressed in tumors of HCC patients; and (2) to determine whether these CTAs are expressed in adjacent macroscopically tumor-free liver tissues of HCC-patients and whether they are an indication of occult metastasis, e.g., by being associated with early recurrence and/or worse HCC-specific survival.

## 2. Patients, Materials, and Methods

This study followed the REMARK (Reporting Recommendations for Tumor Marker Prognostic Studies) guidelines [11].

### 2.1. HCC Patients and Tissues

Ethical approval for this study was granted by the Ethics Committee at Erasmus MC, Rotterdam, the Netherlands, waiving the requirement for informed consent. For the discovery and validation cohorts 100 and 89, respectively, archived surgically-resected fresh frozen HCC-tumor and paired tumor-free liver (TFL) tissue samples (obtained at a distance of > 2 cm from the tumors) were collected after surgery or retrieved from the archives of the Department of Pathology, Erasmus Medical Center Rotterdam. For protein expression analysis 76 formalin-fixed paraffin-embedded (FFPE) paired HCC-tumor and TFL tissues were retrieved from the Dutch nationwide pathology archives (PALGA).

The HCC-patients included in the discovery cohort underwent hepatic resection (*n* = 97 and *n* = 73 for fresh frozen and FFPE samples respectively) or liver transplantation (LTx; *n* = 3 for both fresh frozen and FFPE samples) for HCC in our center between February 1995 and September 2017, and diagnosis of HCC was confirmed by pathological examination. The patients included in the validation cohort underwent hepatic resection (*n* = 89) for HCC in our center between December 2008 and August 2019, and diagnosis of HCC was confirmed by pathological examination.

Medical records were reviewed for clinicopathological variables (listed in Table S1) and the dates of first recurrence, HCC-specific death, and last follow-up on the 1st of September 2019 for the discovery cohort and the 1st of June 2020 for the validation cohort. Date of recurrence was defined as the first date a patient was diagnosed with a LI-RADS5 lesion during radiological follow-up [12]. Local recurrence was defined as intra-hepatic tumor recurrence; all other tumor localizations were classified distant recurrence. Patients that had no recurrence during follow-up or that underwent liver transplantation were censored. HCC-specific death was defined as death due to HCC. Patients that died due to other causes (e.g., postoperative complications, trauma, or other malignancies), did not die during follow-up, or underwent liver transplantation were censored. Time to event was calculated from the day of surgery.

All patients were retrospectively included. Further details of these and other included tissues can be found in the supplementary materials and methods.

### 2.2. Selection of CTAs

**Figure 1:**
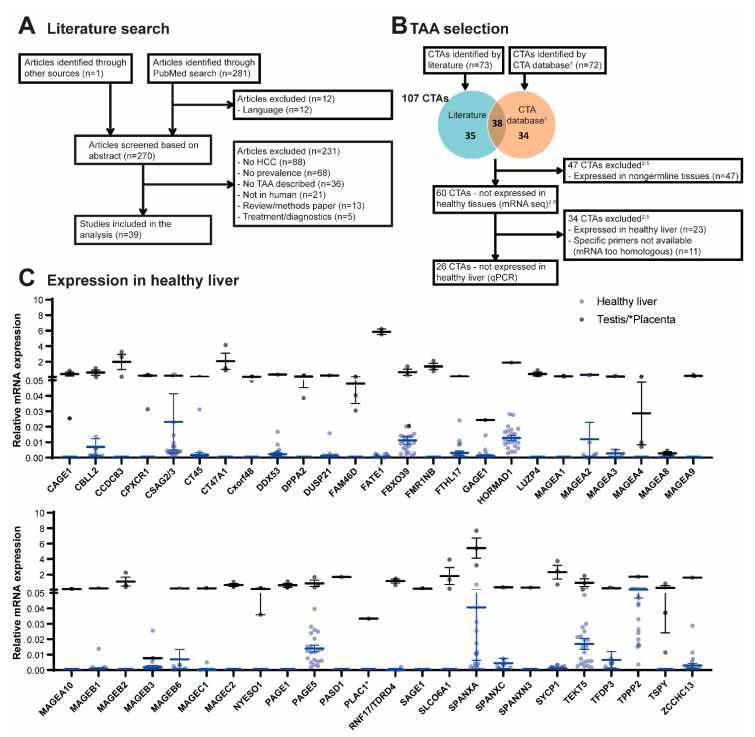
Selection of CTAs. (**A**,**B**) Study Flow Diagram. (**C**) Relative mRNA expression of selected CTAs in healthy donor livers (*n* = 21) in blue and in the respective positive control tissues in black. Control tissues were: placenta (for PLAC1; *n* = 1) or testis (all other CTAs; *n* = 1–3). ^1^ http://www.cta.lncc.br/, accessed on 4 October 2018, ^2^ Hofmann, et al. ^3^ FANTOM consortium, ^4^ HPA consortium, ^5^ GTEx consortium [9,14,15].

A literature search to identify CTAs reported to be expressed in HCC was conducted in PubMed on 4 October, 2018. A summary of this search is provided in Figure 1A and the query in the Supplementary data. Papers written in English that described CTA expression in HCC patients and/or HCC cell lines were included. In addition, the CTA database (http://www.cta.lncc.br/, accessed on 4 October 2018) was consulted to find additional CTAs expressed in HCC and one relevant paper was added [13].

### 2.3. Quantitative Real-Time PCR

RNA was isolated from the frozen tissues and RT-qPCR was performed. The sequences, Tm-values and product lengths of the used primers are provided in Table S2, and detailed methods can be found in the supplementary data file.

### 2.4. Immunohistochemistry

Protein expression was determined by immunohistochemistry (IHC) on tissue microarrays (TMA), that contained three 1 mm cores of each tumor and TFL tissue, as described in the supplementary data file. The stained TMAs were scored blindly by two researchers, based on the intensity of the staining (none, low, intermediate, strong) and the percentage of positive tumor cells or hepatocytes (<10%, 10–50%, 50–90%, >90%). If less than five positive cells per core were observed, the core was scored as 0, and cores smaller than 50% of the original surface were excluded. The final scores were the average scores of the three cores.

### 2.5. Statistical Analysis

All statistical analyses were performed using Graphpad (Version 8.2.1 for Windows, San Diego, CA, USA) and R Statistical software (Version 3.6.1 for Windows, Foundation for Statistical Computing, Vienna, Austria). The correlation analysis was performed in RStudio with the ‘corplot’ package, using Pearson’s correlation coefficient. For creating heatmaps, RStudio was used with the ‘gplots’ and ‘pheatmap’ packages. Survival analysis was performed by the Kaplan–Meier method and the Cox proportional hazards model, using the ‘survminer’ and ‘survival’ packages. Used statistical tests are indicated in the figures. *p*-values < 0.05 were considered significant.

## 3. Results

Selection of 26 CTAs after literature study and exclusion of those expressed in healthy liver.

To determine which CTAs are frequently expressed in HCC tumor tissue, a literature study was conducted. Using a query to identify publications on CTAs expressed in HCC tissue, 281 publication records were obtained through the PubMed search and one relevant paper [13] was added. After removal of non-English publications, 270 publications were screened on title and abstract, of which 231 papers were excluded. Full texts were screened of the remaining 39 studies, which all met the inclusion criteria (Figure 1A). In these 39 studies, expression of 73 different CTAs in HCC was reported: mRNA expression of 51, protein expression of 1, and both mRNA and protein expression of 21 CTAs (Table S3). In addition, the CTA database (http://www.cta.lncc.br/, accessed on 4 October 2018) was consulted, which resulted in identification of 34 different CTAs expressed in HCC; 27 by mRNA, four by protein, and three by protein and mRNA expression. Furthermore, 38 CTAs identified by the CTA database had already been identified in the literature search (Figure 1B). Consecutively, to exclude expression of these 107 CTAs in healthy tissues, studies using next-generation sequencing to quantify mRNA expression levels in samples obtained from a large array of healthy tissues and organs, provided by the FANTOM consortium [14,16], Human Protein Atlas (HPA) consortium [14], and genome-based tissue expression (GTEx) consortium [15], summarized on www.proteinatlas.org, accessed on 4 October 2018, and the genome-wide analysis of CTA mRNA expression by Hofmann et al. [9] were consulted, which led to the exclusion of 47 CTAs expressed in non-germline tissues (Figure 1B).

To verify the absence of expression in healthy adult non-germline tissues, the expression of the remaining 60 CTAs was first determined in 21 healthy livers by RT-qPCR. For 11 CTAs it was not feasible to design specific primers, due to high sequence homology with other genes, and these were excluded. Of the 49 CTAs tested, 23 were expressed in healthy livers, with prevalence rates varying from 14–100%, and therefore also excluded from further analysis. Twenty-four CTAs showed undetectable mRNA expression levels in healthy livers. Two CTAs (*MAGEC1* and *RING finger protein 17* [*RNF17*]) were each found to be expressed in one out of 21 tested healthy livers (with very low relative expression levels of 0.005 and 0.002 respectively), and therefore not excluded (Figure 1C and Table S4). These 26 CTAs were selected for further study.

### 3.1. A Panel of 12 CTAs Is Expressed in More Than 10% of HCC Tumors and Not in Healthy Tissues

**Figure 2:**
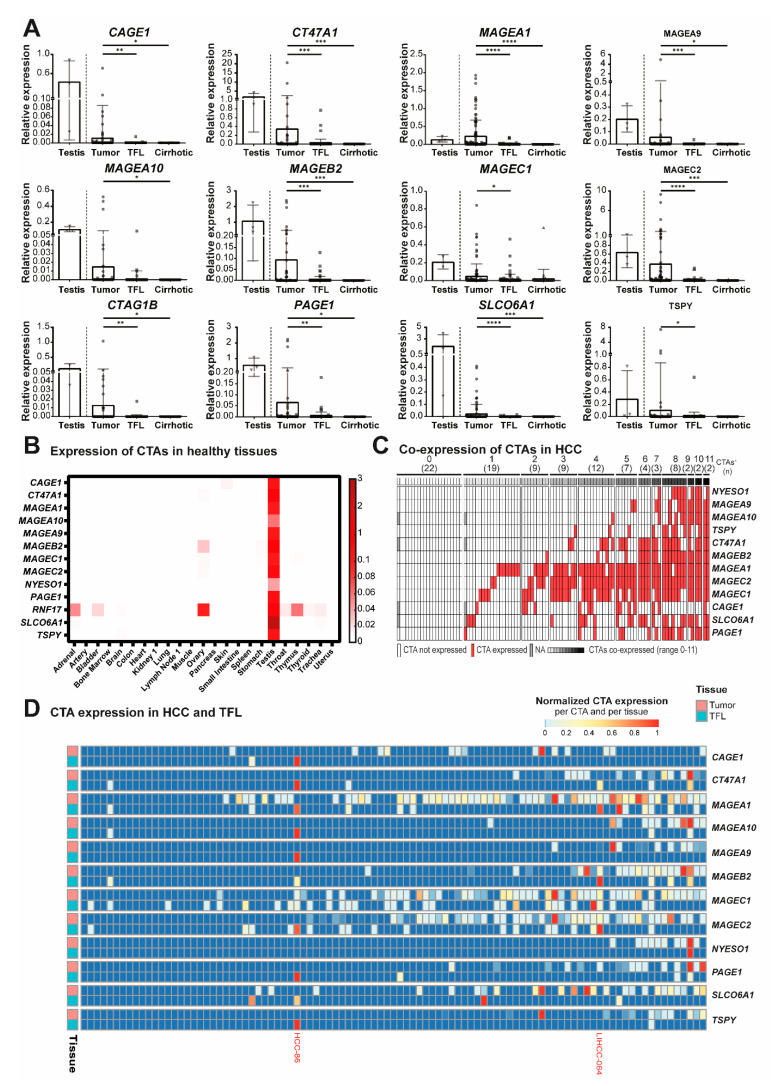
Panel of 12 CTAs expressed in >10% of HCC tumors, but not in healthy tissues. mRNA expression of 12 CTAs in 100 paired HCC and TFL tissues, 35 cirrhotic tissues and 22 different adult healthy tissues, as determined by RT-qPCR. (**A**) mRNA expression of the 12 CTAs that are expressed in more than 10% of HCCs and not in healthy tissues. Dots show individual patient tissues, bars show the mean relative expression level, and error bars show the standard deviation. Wilcoxon signed-rank test, * *p* < 0.05, ** *p* < 0.01, *** *p* < 0.001, **** *p* < 0.0001. (**B**) Heatmap indicating relative mRNA expression levels of all CTAs that are expressed in >10% of HCCs, in healthy adult tissues. (**C**) Heatmap indicating co-expression of CTA mRNA in tumor tissue (**D**) Heatmap of mRNA expression of the 12 CTAs expressed in ≥10% of HCC tumors (rows), in HCC tumors and TFL for every patient (columns). Patients were ordered by number of CTAs expressed in each individual tumor. The –ΔCt values were used and for normalization this data was scaled between 0 and 1 for each CTA in each tissue [((x-(min(x))/(max(x)-min(x)))]. Colors correspond to the value between 0 and 1 and patients LIHCC-064 and HCC-86 are highlighted in red. Heatmap was made in R, using the pheatmap package.

The mRNA expression of these 26 CTAs was determined in 100 paired HCC tumors and TFL and in 35 non-malignant cirrhotic liver tissues. Thirteen CTAs were expressed in tumors of >10% of HCC patients at variable expression levels (Table 1, Figure 2A, and Table S5) and selected for further study. To verify the absence of these 13 CTAs in healthy adult non-germline tissues, and to confirm they are targetable tumor markers, mRNA expression was determined in 23 types of healthy adult tissues other than liver (Figure 2B). Most tissues did not express any CTA, except for ovary which expressed five CTAs. Four CTAs were expressed at very low relative expression levels in ovary (*MAGEB2* 0.002, *cancer/testis antigen family 47 member A1* [*CT47A1*] 0.002, *MAGEC1* 0.003 and *MAGEC2* 0.002). However, *RNF17* had a higher relative expression level (0.097) and was also expressed in other tissues (thyroid, adrenal gland, bladder, brain, throat, trachea, ovary, and thymus), and was therefore excluded from further analysis.

Among the 12 remaining CTAs (Table 1) were 6 members of the MAGE gene family (*MAGEA1*, *MAGEA9*, *MAGEA10*, *MAGEB2*, *MAGEC1*, and *MAGEC2*). *MAGEA1*, *MAGEC1*, and *MAGEC2* were most frequently expressed, with expression rates between 48% and 59% of the tumors. Other CTAs that were expressed in more than 10% of tumors are *cancer antigen 1* (*CAGE1*; 14%), *CT47A1* (27%), *cancer/testis antigen 1B* (*CTAG1B*; 10%), *PAGE family member 1* (*PAGE1*; 18%), *solute carrier organic anion transporter family member 6A1* (*SLCO6A1*; 26%), and *testis-specific Y-encoded protein 1* (*TSPY1*; in 21% of male HCC patients and 0% of female HCC patients, as expected from a gene located on the Y-chromosome) [17].

Thus, based on mRNA expression data, we identified a panel of 12 CTAs prevalently expressed in tumors of HCC-patients, but not in healthy adult tissues except testis. Seventy-eight percent of tumors expressed at least one of these 12 CTAs, 59% expressed at least two CTAs, 50% expressed at least three CTAs, and 40% expressed four or more CTAs (Figure 2C and Figure S1).

### 3.2. CTAs Are Expressed in Tumor-Free Liver Tissues of HCC Patients

To investigate whether CTA expression in TFL could be an indication of occult metastasis, the expression of these CTAs was also determined in TFL. Despite the TFL being located at least 2 cm away from the tumor and being classified as tumor-free by a pathologist, all 12 CTAs were expressed in these tumor-free liver tissues of HCC patients, although at significantly lower levels (Table 1, Figure 2A and Table S5). Forty-five percent of patients expressed at least one CTA in TFL (Figure S1). The CTAs most frequently expressed in TFL were *MAGEA1* (13% of patients), *MAGEC1* (32%) and *MAGEC2* (19%). The latter two were also found to be expressed in approximately 25% of cirrhotic liver tissues of HCC-patients without liver cancer, suggesting that their expression may be activated during early (pre-)malignant transformations in the liver. Interestingly, when a particular CTA was detected in TFL, it was often also present in the tumor (Figure 2D); 85% of patients that expressed any CTA in TFL also had CTA expression in their tumor. For example, LIHCC-064 expressed seven CTAs in their tumor, of which five were also expressed in TFL, suggesting that CTA-expressing cells in TFL were derived from the primary tumor.

### 3.3. CTAs Are Expressed on Protein Level in HCC Tumors and TFL

Consecutively, we examined protein expression of these CTAs in tumor and TFL tissues of 78 HCC-patients of which FFPE blocks were available (patient characteristics are shown in Table S6). Protein expression of MAGEA1, MAGEA10, MAGEC1, MAGEC2, and NYESO1 in HCC tumors has previously been reported by our group [18]. For CAGE1 no suitable IHC antibodies (Ab) are available. The MAGEB2 IHC Ab showed reliable staining in testis tissue; however, we could not detect any positive cells in HCC and TFL tissues. TSPY1 and SCLO6A1 Abs demonstrated an unspecific staining pattern and a punctate staining that did not allow for quantification of positive cells, respectively, and were therefore discarded (Figure S2) [17].

**Figure 3:**
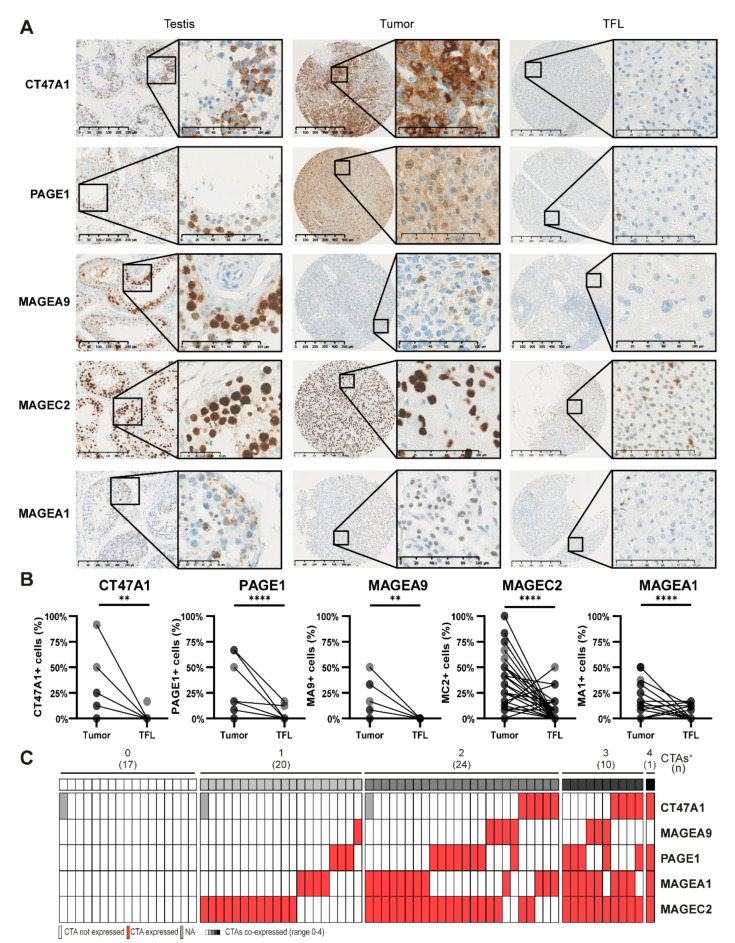
Proteins CT47A1, PAGE1, MAGEA9, MAGEC2, and MAGEA1 are expressed in HCC tumors and TFL. TMAs of tumor and TFL tissues were immunohistochemically stained to study the protein expression of aforementioned CTAs. (**A**) Representative examples of immunohistochemical stains in testis, a positive HCC tumor tissue and TFL tissue. (**B**) Scores of percentages of tumor cells or hepatocytes expressing CT47A1, PAGE1, MAGEA9, MAGEC2, and MAGEA1 in tumors and paired TFL (*n* = 78). Average scores of three tissue cores are shown. Wilcoxon signed-rank test, ** *p* < 0.01, **** *p* < 0.0001. (**C**) Heatmap indicating co-expression of CTA-proteins in tumor tissue. TMA slides were scanned by a Nanozoomer (Hamamatsu, Hamamatsu, Japan), and analyzed by NDP.view2 software (Hamamatsu).

CT47A1, PAGE1, MAGEA9, MAGEC2, and MAGEA1 were detected at protein level in tumor tissues (CT47A1 in 14%, PAGE1 in 23%, MAGEA9 in 11%, MAGEC2 in 59% and MAGEA1 in 34% of tumors; Figure 3, Figure S3). These CTAs were expressed by tumor cells, similar to MAGEA10, MAGEC1, and CTAG1B proteins as demonstrated in our previous study [18]. Seventy-one percent of HCC tumor tissues expressed at least one of these CTAs on protein level (Figure 3C). In the majority of patients, only part of the tumor cells expressed these CTAs. Proportions of tumor cells which expressed these CTAs were variable between different patients (Figure 3B), similar to expression intensity (Figure S3). MAGEA9 was not expressed in any TFL tissue, while we observed expression of CT47A1, PAGE1, MAGEC2, and MAGEA1 in single scattered hepatocytes in 1%, 3%, 17%, and 9% of TFL tissues respectively (Figure 3B and Figure S3). Thirty percent of patients expressed at least one CTA protein in their TFL tissue. Most CTA protein expression was focal, as illustrated by the observation that in most patients only part of the tumor cores included in the TMA showed protein expression (Figure S4).

In conclusion, the CTAs that were studied for protein expression, also showed protein expression in tumors and, except for MAGEA9, also in TFL.

### 3.4. CTA Expression in TFL Is Correlated with Early HCC Recurrence and HCC-Specific Survival after Surgical Resection

**Figure 4:**
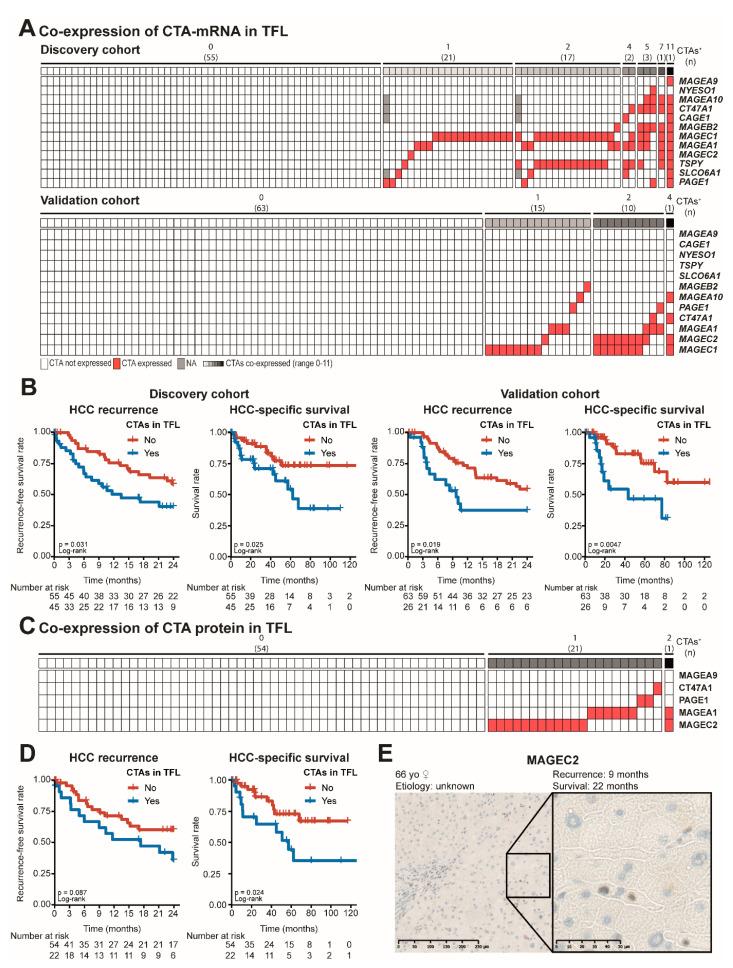
Both mRNA and protein expression of CTAs in TFL are associated with HCC recurrence and HCC-specific survival. (**A**) Heatmap indicating co-expression of CTA mRNA in tumor-free liver tissue in the discovery and validation cohort. (**B**) Early HCC recurrence and HCC-specific survival in HCC patients by CTA mRNA expression in TFL in the discovery and validation cohort. Plus-signs indicate censored data. Cox–Mantel log-rank test. (**C**) Heatmap indicating co-expression of CTA protein in tumor-free liver tissue. (**D**) Early HCC recurrence and HCC-specific survival in HCC patients by CTA protein expression in TFL. Plus-signs indicate censored data. Cox–Mantel log-rank test. (**E**) Representative example of IHC staining of MAGEC2 protein expression in TFL and accompanying patient data. TMA slides were scanned by a Nanozoomer (Hamamatsu), and analyzed by NDP.view2 software (Hamamatsu).

To determine whether CTA expression in TFL could be an indication of occult micrometastasis, we analyzed its association with early HCC recurrence, defined as HCC recurrence within 2 years, and HCC-specific survival. Expression of CTA mRNA in TFL (Figure 4A) was negatively associated with both early HCC recurrence and HCC-specific patient survival after surgical resection (Figure 4B and Figure S5). Early recurrence was observed in 64% of patients with CTA expression in TFL versus 40% in those without. Two-year HCC-specific survival rates were 71% and 89% in patients with and without CTA expression in TFL, respectively. These results were confirmed in a validation cohort, consisting of 89 HCC patients. In this cohort 29% of HCC patients expressed one or more CTAs in TFL, with a maximum of four CTAs. Early recurrence was observed in 54% of patients with CTA expression in TFL versus 38% in those without. Two-year HCC-specific survival rates were 69% and 94% in patients with and without CTA expression in TFL, respectively. Interestingly, when we discriminated between local intra-hepatic and distant cancer recurrence, patients with CTA-expression in TFL showed more and faster intra-hepatic cancer recurrence in both cohorts. In contrast, there was no difference in distant cancer recurrence between patients with or without CTA-expression in TFL in the discovery cohort, while in the validation cohort the difference in distant cancer recurrence between patients with and without CTA-expression in TFL was small and borderline significant (*p* = 0.046) (Figure S6). The robust association with intra-hepatic recurrence in both cohorts supports the hypothesis that CTA-expression in TFL may reflect the presence of histologically non-distinguishable intra-hepatic micro-metastases.

Patients with and without CTA-expression in TFL did not differ in any clinical or histological characteristic, including type of surgery, tumor differentiation grade, and vascular invasion (Table S1; all *p*-values > 0.05). In multivariate analysis, mRNA expression in TFL was an independent prognostic factor for early HCC recurrence (hazard ratio (HR) 2.3 and 2.1, for the discovery and validation cohort respectively) and HCC-specific survival (HR 2.3 and 3.6, respectively) in both cohorts, as is shown in Table 2. CTA protein expression in TFL (Figure 4C) was associated with poor postsurgical outcome as well (Figure 4D). In multivariate analysis CTA protein expression in TFL was also an independent prognostic factor for HCC recurrence (HR 2.5) and HCC-specific survival (HR 3.8; Table S7). An example of CTA protein expression in TFL is shown in Figure 4E, the MAGEC2 expressing cells were scattered across the TFL. All TFL tissues with CTA expression were reassessed by a medical pathologist to verify the absence of histologically detectable HCC metastasis. Except for extensive vascular invasion in one patient, which also expressed PAGE1 in TFL (Figure S7), no histological indications for the presence of malignant cells in TFL were present. Notably, both survival analysis and cox-regression analysis of CTA expression in tumor tissues did not show associations with postsurgical outcome (Table S8 and Figure S8).

In conclusion, we found that CTA expression in TFL is an independent negative prognostic factor of both HCC recurrence and HCC-specific survival, and we validated these findings in a validation cohort. This may indicate that occult CTA-expressing (pre-)malignant cells are present in the remaining liver tissue after tumor resection and that these cells could be responsible for HCC recurrence, especially for intra-hepatic recurrence, after surgery.

## 4. Discussion

We established a novel panel of 12 CTAs, each expressed in at least 10% of HCC tumors and not in healthy tissues except immune-privileged testis. Based on mRNA analysis, approximately 80% of HCC-patients expressed one or more of these antigens in their tumor tissues, whereas protein expression of five of these CTAs was detected in approximately 70% of HCC tumors. In addition, we found that 45% of HCC-patients included in the discovery cohort expressed one or more of the 12 CTAs of our panel in their histologically tumor-free liver tissue, which was associated with early HCC recurrence and worse patient survival after curative surgery. These associations were confirmed in a validation cohort, in which 29% of HCC patients expressed one or more CTAs in TFL.

High recurrence rates after surgery with curative intent worsens the survival of HCC patients. Aufhauser, et al. [19] hypothesized that early recurrence, defined as recurrence within 2 years after tumor resection, is due to occult metastasis rather than de novo tumor formation, but failed to find biomarkers identifying occult metastasis at the time of resection. Therefore, we aimed to find biomarkers detecting occult multifocality at the time of resection, in order for these patients to be selected for adjuvant treatment. We hypothesized that biomarkers identifying occult multifocality should be abundantly and relatively frequently expressed in tumor tissues, to allow for high sensitivity, and should be completely absent in healthy tissues, to allow for high specificity.

CTA expression in tumors of HCC-patients has been studied before; however, as demonstrated by the results of our literature study (Table S3), most studies investigated only a few CTAs, determined either RNA or protein expression but not both, did not exclude CTAs expressed in healthy tissues, and most notably, did not look at or acknowledge CTA expression in tumor-free liver (Figure 1B,C, Table S5). Thus, to assure we would determine the CTAs most likely to serve as markers for occult multifocality in TFL, we repeated CTA expression analysis in tumor tissues and confirmed absence of the selected CTAs in healthy tissues. As far as we are aware, the present study is the most comprehensive investigation of CTA-expression in tumor and paired TFL tissues of HCC-patients performed. Another recent report used the GEPIA database to analyze CTA expression in tumors of HCC patients, but did not investigate CTA expression in non-cancerous liver tissues of HCC patients [20]. An additional benefit of excluding CTAs expressed in healthy tissues would be their suitability for therapeutic targeting, as targeting proteins exclusively expressed in the tumor will not lead to therapy-induced auto-immunity in potential future clinical applications [8].

As the expression of CTAs in tumor-free (liver) tissues of patients with HCC or other cancer types has barely been investigated before, their association with cancer recurrence or patient survival has also not been investigated in HCC, nor any other types of cancer. Therefore it was unknown if CTAs could serve as biomarkers for occult multifocality. The 2-year recurrence rate of 46% in the study by Aufhauser, et al. [19] is comparable to the observed rate of 50% and 43% in the discovery and validation cohorts of this study, respectively, and therefore we expect the cohorts to be comparable. Unexpectedly, we observed mRNA expression of one or more of the 12 CTA’s of our panel in histologically tumor-free liver tissues in a substantial percentage of patients; 45% of tumor-free tissues included in the discovery cohort and in 29% of tumor-free tissues in the validation cohort. Protein expression of one or more of four of these CTAs was detected in non-cancerous liver tissues of 40% of patients of the discovery cohort. The 2-year recurrence rates in our cohorts were significantly higher in patients with CTA mRNA-expression in TFL compared to patients without CTA-expression in TFL; 64% vs 40% in the discovery cohort and 54% vs 38% in the validation cohort. Interestingly, in both cohorts CTA expression in TFL was associated with local intra-hepatic recurrence, but in the discovery cohort not with distant cancer recurrence. Moreover, CTA mRNA expression profiles in TFL were generally similar to those in the corresponding tumors, and our preliminary immunohistochemical data show that CTA-expressing cells in TFL were either single cells or small foci. Based on these observations, we hypothesize that CTA-expressing cells in TFL of patients with early intra-hepatic HCC recurrence indeed represent occult intra-hepatic micro-metastases, and are less likely to represent de novo tumors. This hypothesis is supported by a study performed in colorectal cancer patients with liver metastasis [21]. In TFL, they detected low frequencies of somatic mutations that were also observed in matched tumor samples, despite normal histological appearance. Since these mutations were not found in the matched blood samples, it was hypothesized that tumor DNA or tumor cells diffused or migrated into the surrounding normal tissue [21]. However, the authors did not correlate this to either cancer recurrence or survival. Similarly, a previous study detected MAGE-antigen expression in lung tissues of former smokers at risk for NSCLC development [22], but also did not show any data regarding actual NSCLC development.

Determining lymph node involvement is a widely accepted method for staging a wide variety of cancers. The lymph node metastases themselves are not the cause of death in most patients; however, lymph node involvement is correlated with the spread to vital organs [23]. Therefore, it is correlated with reduced patient survival and an important prognostic factor [24,25]. Likewise, we showed that CTA expression in tumor-free tissue is correlated with recurrence of HCC after curative surgery, independent of conventional prognostic factors. We hypothesize that these CTAs in TFL are expressed by micro-metastases, leading to tumor recurrence and eventually HCC-related death. Detection of occult metastasis in tumor-free tissue, by detection of CTA expression or other methods such as mutation analysis, could be used as a new concept to identify patients at risk for developing (local) metastasis. Moreover, these CTAs could serve as targets to prevent and/or treat these (micro-)metastases.

One way to target these CTAs is by vaccination. Most therapeutic cancer vaccination studies targeting CTAs have been performed in advanced cancer patients with high tumor load in which an immunosuppressive tumor microenvironment has been established, and showed modest clinical results [26]. Based on our data showing the presence of scattered single CTA-expressing cells and small foci of CTA-expressing cells in TFL of 29–45% of resected HCC-patients, therapeutic vaccination with CTAs after tumor resection might be a promising approach to prevent HCC recurrence in such patients. Compared to vaccination in advanced cancer, we expect that the low tumor load remaining after resection of detectable tumors may enhance the probability of effective immunological eradication of CTA-expressing (pre-)malignant cells. A prerequisite for therapeutically targeting antigens by vaccination, is that the antigens are immunogenic. Most of the CTAs included in our panel have previously been proven immunogenic in cancer patients [27]. More specifically in HCC patients, we and other research groups have demonstrated the presence of MAGEA1-, MAGEA10-, MAGEC2-, and NY-ESO-1-specific T-cells, both in blood and in tumors [28,29,30,31,32]. In addition, NY-ESO-1 and TSPY-specific IgG have been detected in HCC-patients [33,34], while CT47A1-, PAGE1-, and SLCO6A1-specific antibodies were recently detected in NSCLC patients [35].

Several CTAs of our panel, such as the MAGE-family members, *CTAG1B*, *TSPY1*, and *CAGE1*, are functionally involved in tumorigeneses and cancer progression by modulating gene expression, regulating mitosis, and tumorigenic signaling [8,10,36,37,38]. More specifically, MAGE-A9 and TSPY have been shown to be involved in HCC tumor cell proliferation [36,38]. Their role in cancer progression is further supported by data showing that CTA expression is more prevalent in advanced tumors [39,40]. Importantly, the involvement of these CTAs in cancer progression may prevent antigen loss upon therapeutic targeting [37].

We acknowledge several limitations of this study. First, since the etiologies of HCC differ geographically, this CTA-panel might not be applicable to non-Western HCC-populations. Secondly, future research is required to investigate whether CTA-expressing cells in TFL are really (pre-)malignant cells that can give rise to cancer recurrence. Moreover, as not all HCC tumors expressed the selected CTAs, occult micro-metastasis of the tumors not expressing CTAs may be missed. Finally, for the concept–detection and treatment of occult multifocality by detection of therapeutically targetable CTAs in supposedly tumor-free tissues—to become widely applicable, it should be validated in other cancer types.

## 5. Conclusions

We established a panel of 12 testis-restricted CTAs that are expressed in tumors of almost 80% of HCC patients. In addition, we demonstrated expression of these CTAs in tumor-free liver tissues of 45% and 29% of HCC-patients in two different cohorts. The negative association between expression of these CTAs in TFL and HCC-recurrence and patient survival, independent of clinical and histological tumor characteristics, combined with immunohistochemisry data showing scattered CTA-expressing cells in TFL, suggests that CTA-expressing (pre-)malignant cells remain present in the liver after tumor resection, and are indicative for the potential relevance of therapeutic targeting of these antigens. To prevent tumor recurrence, HCC patients with CTA expression in TFL could be selected for adjuvant therapy, either by therapeutic targeting of these CTAs, other (immuno-) therapeutic strategies, or a combination of both.

## Figures and Tables

**Table 1:** mRNA expression of CTAs in tumor and TFL of HCC-patients. **^1^** Percentage of hepatocellular carcinomas (HCC) expressing mRNA of the CTA–meaning a Ct-value <35 and relative expression >0.001 (*n* = 100); ^2^ Mean relative expression (relative to the geometric mean of the 3 household genes- GUSB, HPRT1, PMM1) level in HCCs expressing the CTA and range; ^3^ Mean relative expression of the CTA in HCC expressing the CTA, relative to the relative mean expression in 3 testis tissues; ^4^ Percentage of paired tumor-free liver (TFL) tissues expressing mRNA of the CTA (*n* = 100); ^5^ Mean relative expression level in TFLs expressing the CTA and range; ^6^ Mean relative expression of the CTA in TFL expressing the CTA, relative to the relative mean expression in 3 testis tissues; ^7^ Percentage of non-cancerous/non-dysplastic cirrhotic liver tissues expressing the CTA (*n* = 35); * % in male.

CTA	mRNA-Positive HCC (%) ^1^	Mean in mRNA-+ HCC (Range) ^2^	Relative Expression HCC (Compared to Testis) ^3^	mRNA-Positive TFL (%) ^4^	Mean in mRNA-+ TFL (Range) ^5^	Relative Expression TFL(Compared to Testis) ^6^	mRNA-Positive Cirrhotic Tissue ^7^
*CAGE1*	14.4	0.082 (0.003–0.711)	0.188	2.0	0.009 (0.003–0.015)	0.020	0
*CT47A1*	26.8	1.311 (0.001–20.565)	0.632	6.1	0.255 (0.01–0.769)	0.123	0
*MAGEA1*	58.6	0.403 (0.003–1.926)	4.170	13.0	0.055 (0.005–0.188)	0.567	0
*MAGEA9*	14.1	0.41 (0.001–4.953)	2.848	1.0	0.035 (0.035–0.035)	0.243	0
*MAGEA10*	12.4	0.123 (0.002–0.518)	1.080	4.1	0.028 (0.004–0.088)	0.249	0
*MAGEB2*	24.2	0.395 (0.002–2.4)	0.761	6.0	0.053 (0.018–0.127)	0.102	0
*MAGEC1*	47.5	0.109 (0.001–0.841)	0.407	32.0	0.047 (0.002–0.466)	0.174	28.6
*MAGEC2*	55.6	0.692 (0.001–9.305)	1.542	19.0	0.041 (0.003–0.28)	0.091	25.7
*NYESO1*	10.1	0.13 (0.007–1.04)	0.525	1.0	0.018 (0.018–0.018)	0.071	0
*PAGE1*	18.2	0.37 (0.002–2.225)	1.001	5.0	0.059 (0.009–0.179)	0.159	2.9
*SLCO6A1*	25.8	0.095 (0.002–0.411)	0.053	4.1	0.011 (0.004–0.017)	0.006	2.9
TSPY *	21.0	0.827 (0.004–7.401)	34.135	4.8	0.218 (0.001–0.641)	9.012	4.2

**Table 2:** CTA mRNA-expression in TFL is an independent prognostic factor of HCC recurrence and HCC-specific survival. Univariate and multivariate analyses of factors associated with recurrence and survival according to the cox proportional hazard model. Abbreviations: AFP, alphafoetoprotein; 95% CI, 95% confidence interval; CTA, cancer testis antigen; HR, hazard ratio; TFL, tumor-free liver. Significant values (*p* < 0.05) are indicated in bold.

Discovery Cohort								
	Early Recurrence (<2 yr)				HCC-Specific Survival			
	Univariate Analysis		Multivariate Analysis		Univariate Analysis		Multivariate Analysis	
Variable	HR (95% CI)	*p*-Value	HR (95% CI)	*p*-Value	HR (95% CI)	*p*-Value	HR (95% CI)	*p*-Value
≥1 CTA in TFL	2.3 (1.3–4.0)	**0.0034**	2.5 (1.47–4.5)	**0.003**	2.4 (1.1–5.4)	**0.03**	2.3 (1.0–5.3)	**0.044**
≥2 CTAs in TFL	2.1 (1.2–3.7)	**0.013**			1.7 (0.7–3.9)	0.22		
≥3 CTAs in TFL	4.2 (1.9–9.4)	**0.00053**			5.1 (1.9–14)	**0.0015**		
Number of CTAs in TFL (numeric)	1.3 (1.2–1.5)	**2.0 × 10^−5^**			1.3 (1.1–1.5)	**0.0011**		
>1 tumor	1.2 (0.7–2.0)	0.56			1.1 (0.5–2.4)	0.83		
>2 tumors	2.6 (1.3–4.9)	**0.0042**	2.4 (1.2–4.7)	**0.02**	1.8 (0.7–4.9)	0.22		
Cirrhosis	1.6 (0.9–2.8)	0.12			1.5 (0.7–3.4)	0.33		
Chronic viral hepatitis	2.3 (1.3–4.0)	**0.0031**	2.7 (1.5–5.0)	**0.001**	3.3 (1.5–7.2)	**0.0032**	4.63 (2.0–10.8)	**0.0004**
Vascular invasion	1.3 (0.7–2.3)	0.41			2.2 (0.96–4.9)	0.063		
Tumor > 5 cm	1.3 (0.7–2.3)	0.37			2.3 (0.9–5.7)	0.081		
AFP > 200 ug/L	1.9 (1.0–3.4)	**0.034**			2.7 (1.2–6)	**0.013**		
AFP > 400 ug/L	2.4 (1.3–4.5)	**0.0051**	3.0 (1.5–5.8)	**0.001**	3.3 (1.5–7.3)	**0.0038**	4.0 (1.7–9.4)	**0.002**
**Validation Cohort**								
	**Early Recurrence (<2 yr)**				**HCC-Specific Survival**			
	**Univariate Analysis**		**Multivariate Analysis**		**Univariate Analysis**		**Multivariate Analysis**	
**Variable**	**HR (95% CI)**	***p*-value**	**HR (95% CI)**	***p*-value**	**HR (95% CI)**	***p*-value**	**HR (95% CI)**	***p*-value**
≥1 CTA in TFL	2.2 (1.1–4.2)	**0.022**	2.1 (1.1–4.1)	**0.03**	3.3 (1.4–7.7)	**0.0074**	3.6 (1.5–8.8)	**0.004**
≥2 CTAs in TFL	1.5 (0.58–3.8)	0.41			2.3 (0.83–6.3)	0.11		
≥3 CTAs in TFL	1.1 × 10^−7^ (0-Inf)	1			3.9 × 10^−8^ (0-Inf)	1		
Number of CTAs in TFL (numeric)	1.2 (0.89–1.7)	0.21			1.4 (0.95–2)	0.095		
>1 tumor	2.1 (1–4.2)	**0.043**	2.2 (1.1–4.5)	**0.03**	0.9 (0.27–3.1)	0.87		
>2 tumors	1.7 (0.67–4.4)	0.26			0.96 (0.22–4.1)	0.96		
Cirrhosis	0.77 (0.39–1.5)	0.45			2.3 (0.97–5.5)	0.059	2.6 (1.1–6.3)	**0.03**
Chronic viral hepatitis	0.91 (0.42–2)	0.82			0.98 (0.36–2.7)	0.97		
Vascular invasion	2.1 (0.98–4.4)	0.055			1.5 (0.59–3.9)	0.38		
Tumor > 5 cm	2.5 (1.2–5)	**0.011**	2.6 (1.3–5.3)	**0.007**	1.5 (0.61–3.6)	0.38		
AFP > 200 ug/L	1.6 (0.8–3.3)	0.18			0.83 (0.28–2.5)	0.74		
AFP > 400 ug/L	1.3 (0.61–2.9)	0.46			0.65 (0.19–2.2)	0.5		

## Data Availability

Data is available upon reasonable request

## Supplementary Materials

The following are available online at https://www.mdpi.com/article/10.3390/cancers13102499/s1, Table S1: Patient characteristics of HCC-patients in discovery and validation cohort based on CTA expression in TFL, Table S2: Primer sequences and annealing temperatures (Tm) used for RT-qPCR, Table S3: Results of the literature search and overview of included articles, Table S4: Frequency table of healthy liver tissues (*n* = 21) expressing mRNA of the CTAs, Table S5: Expression of excluded CTAs in HCC patients and in cirrhotic liver tissues without malignancy, Table S6: Patient characteristics of HCC-patients included in protein expression analysis, Table S7: Cox regression analysis of HCC recurrence and HCC-specific survival based on CTA protein expression in TFL, Table S8. Cox regression analysis of HCC recurrence and HCC-specific survival based on CTA mRNA expression in HCC tumors, Table S9: Antibodies used for immunohistochemistry, Figure S1: Number of CTAs co-expressed in HCC tumors and TFL, based on mRNA expression, Figure S2: TSPY expression in female HCC tumors and SLCO6A1 expression, Figure S3: Protein expression of CTAs in HCC tumors paired tumor free liver, Figure S4: Proteins are focally expressed in most tumors, Figure S5. HCC recurrence and HCC-specific survival by number of CTAs expressed in the discovery cohort (based on mRNA expression) in TFL, Figure S6: Local and distant HCC recurrence by expression of CTAs in TFL in the discovery and validation cohort, Figure S7: PAGE1 expressing tumor nodule in TFL, Figure S8: HCC recurrence and HCC-specific survival by CTA mRNA-expression in tumor tissue in the discovery cohort.

## Abbreviations

AFP: alpha-foetoprotein; CAGE1, cancer antigen 1; CPI, checkpoint inhibition; CTAG1B, cancer/testis antigen 1B (also known as NYESO1 [New York esophageal squamous cell carcinoma 1]); CT47A1, cancer/testis antigen family 47 member A1; CTA, cancer testis antigen; FFPE, formalin-fixed paraffin-embedded; HBV, hepatitis B virus; HCC, hepatocellular carcinoma; HCV, hepatitis C virus; HR, hazard ratio; IHC, immunohistochemistry; MAGE, melanoma antigen; NAFLD, non-alcoholic fatty liver disease; NSCLC, non-small cell lung cancer; PAGE1, PAGE family member 1; RNF17, RING finger protein 17; SLCO6A1, solute carrier organic anion transporter family member 6A1; TFL, tumor-free liver; TMA, tissue microarray; TSPY, testis-specific Y-encoded protein 1.
