# Supplementary material for: Expression of Cancer Testis Antigens in Tumor-Adjacent Normal Liver Is Associated with Post-Resection Recurrence of Hepatocellular Carcinoma
Source: Cancers (Basel). 2021 May 20;13(10):2499. doi: 10.3390/cancers13102499 (PMC8160719; doi:10.3390/cancers13102499)
Supplement: Supplementary file 1 [file cancers-13-02499-s001.zip › cancers-1145638-supplementary.pdf]

# Supplementary Materials:

# Expression of Cancer Testis Antigens in Tumor-Adjacent Normal Liver is Associated with Post-Resection Recurrence of Hepatocellular Carcinoma

**Materials & Methods**

*Liver and healthy tissue samples*

Cirrhotic and healthy (liver) tissues

Freshly frozen healthy liver tissues (n=21) were obtained during liver transplantation from donor liver grafts at the end of cold ischemic storage. Archived freshly frozen tissue samples of non-cancerous cirrhotic livers (n=35) were retrieved from the tissue bank of the Department of Pathology, Erasmus Medical Center Rotterdam. The non-cancerous cirrhotic liver tissues had been retrieved from patients who underwent liver transplantation for liver cirrhosis in our center between May 2007 and June 2017. The etiology of the cirrhosis was determined by information from medical records, laboratory tests and pathological examination of the explanted livers. Cirrhotic livers with malignancies, diagnosed by pathological examination, were excluded.

RNA isolated from fresh frozen healthy adrenal gland (R1234004-50), artery (HR-810), brain (R1234035-50), colon (R1234090-50), heart (R1234122-50), lung (R1234152-50), muscle (R1234171-50), ovary (HR-406), pancreas (R1234188-50), skin (R1234218-50), small intestine (R1234226-50), stomach (HR-302), testis (R1234260-50), throat (R1234263-10), thymus (HR-702), thyroid (R1234265-50), trachea (R1234160-50), urinary bladder (R1234010-50) and uterus (R1234274-50) tissues were purchased from AMS Biotechnology Ltd, Abingdon, UK. Bone marrow derived from a healthy donor (Department of Hematology, Erasmus MC), healthy kidney tissue obtained from a donor kidney (Department of Internal Medicine, Erasmus MC) and RNA of healthy testis tissues (Department of Pathology, Erasmus MC) were kindly provided. Lymph node and spleen tissues were collected from samples retrieved during liver transplantation in our center in September 2019.

*Quantitative real-time PCR*

RNA was isolated using the NucleoSpin® RNA isolation kit of Macherey-Nagel (Dueren, Germany) according to manufacturer's instructions. RNA quality was assessed with NanoDrop 2000 (Thermo Scientific), using 260/230 ratios. Samples with a ratio <2.0 were excluded and if possible RNA was re-isolated. RNA (4 ug) was reverse-transcribed into cDNA using PrimeScript™ RT master Mix (Perfect Real Time, Takara, cat# RR036A), according to the manufacturer's instructions. RT-qPCR was performed using SYBR™ Green PCR Master Mix (ThermoFisher) in a StepOnePlus™ Real-Time PCR System (Applied Biosystems), using 12.5 ng cDNA per reaction, with the following conditions: 50°C for 2 minutes, 95°C for 2 minutes, then 38 cycles of 95°C for 15 seconds, 58-62°C for 15 seconds (according to the Tm of the primers), 72°C for 1 minute, and then finally for the Melt Curve stage 95°C for 15 seconds, 60°C for 1 minute and a 0.7°C step-wise increase until 95°C was reached. All Ct-values over 35 were considered negative. The level of target gene expression relative to the geometric mean of three control genes (HPRT1, GUSB, PMM1) [1] was calculated by $2^{-\Delta\Delta T}$ method, after which a cut-off of 0.001 was used to define expression. All amplifications were performed in at least two technical repeats. Means of technical replicates were used for analysis. Primers were designed with Primer Blast (NCBI), efficiency was determined by dilution of cDNA and product length was determined by gel electrophoresis.

*Immunohistochemistry*

The FFPE blocks of the HCC and TFL tissues were examined by a pathologist (MD) to mark tumor and tumor-free liver tissues. A TMA Grand Master (2.5; 3D Histech) was used to create tissue microarrays (TMA). Three tissue cores of 1 mm were taken of each tissue and placed in a recipient formalin block. Immunohistochemistry (IHC) was performed using an automated, validated and accredited staining system (Ventana Benchmark ULTRA, Ventana Medical Systems, Tucsen, AZ, USA) using the optiview universal DAB detection Kit (cat.760-700, Ventana Medical Systems). In brief, following deparaffinization and heat-induced antigen retrieval tissue sections were incubated with each of the primary antibodies according to their optimized incubation time and concentration **(Table S9)**. The antibodies were titrated using testis as a positive control tissue and placenta and spleen as negative control tissues. Incubation was followed by hematoxylin II counter stain for 12 minutes and then a blue colouring reagent for 8 minutes according to the manufacturer's instructions (Ventana Medical Systems, Tucsen, AZ, USA). The stained TMAs were then scanned using a Nanozoomer (Hamamatsu), and analyzed using NDP.view2 software (Hamamatsu).

**Search query:**

(("cancer testis antigen"[All Fields] OR ((("cancer"[All Fields] OR "neoplas*"[All Fields]) AND ("testis"[All Fields] OR "testes"[All Fields]) AND ("Antigens, Neoplasm"[Majr] OR "antigen*"[All Fields] OR "Ags"[All Fields] OR "ag"[All Fields] OR "gene"[All Fields] OR "genes"[All Fields] OR "antigen*"[All Fields])))) AND (("Carcinoma, Hepatocellular"[Majr]) OR "Fibrolamellar hepatocellular carcinoma" [Supplementary Concept] OR "liver cell carcinoma"[All Fields] OR "liver cancer"[All Fields] OR "hepatocellular carcinoma cell line"[All Fields] OR (("liver"[All Fields] OR "hepat*"[All Fields]) AND ("carcinoma*"[All Fields] OR "ca"[All Fields] OR "cas"[All Fields] OR "cancer*"[All Fields])) OR "hepatocarcinom*"[All Fields])) AND "Humans"[Mesh]

Pubmed search 04-10-2018

**Table S1.** Patient characteristics of HCC-patients in discovery and validation cohort based on CTA expression in TFL.

| Characteristic | Discovery cohort CTA in TFL- (n=55) | Discovery cohort CTA in TFL+ (n=45) | Validation cohort CTA in TFL – (n=63) | Validation cohort CTA in TFL+ (n=26) |
|---|---|---|---|---|
| **Age at surgery (years)** | | | | |
| Mean ± SD | 60.0 ± 14.3 | 59.8 ± 15.0 | 65.9 ± 10.7 | 60.4 ± 11.0 |
| Median (range) | 63 (11-82) | 64 (16-80) | 67 (34-85) | 61 (36-76) |
| **Sex – no. (%)** | | | | |
| Male | 36 (65.5) | 27 (60) | 49 (77.8) | 15 (57.7) |
| Female | 19 (34.5) | 18 (40) | 14 (22.2) | 11 (42.3) |
| **Race – no. (%)** | | | | |
| White | 47 (85.5) | 36 (80) | 52 (82.5) | 20 (76.9) |
| African | 3 (5.5) | 5 (11.1) | 4 (6.3) | - |
| Asian | 4 (7.3) | 4 (8.9) | 5 (7.9) | 5 (19.2) |
| Not reported | 1 (1.8) | - | 2 (3.2) | 1 (3.8) |
| **Etiology – no. (%)** | | | | |
| No known liver disease | 14 (25.5) | 19 (42.2) | 19 (30.2) | 7 (26.9) |
| Alcohol | 16 (29.1) | 5 (11.1) | 11 (17.5) | 8 (30.8) |
| Hepatitis B | 8 (14.5) | 4 (8.9) | 10 (15.9) | 3 (11.5) |
| NASH | 5 (9.1) | 3 (6.7) | 12 (19.0) | 4 (15.4) |
| Hepatitis C + Alcohol | 3 (5.5) | 5 (11.1) | - | - |
| Hepatitis B + Alc/HepC/HepD/NASH | 2 (3.6) | 4 (8.9) | 1 (1.6) | 1 (3.8) |
| Hepatitis C | 4 (7.3) | 2 (4.4) | 5 (7.9) | 1 (3.8) |
| Fibrolamellar HCC | 2 (3.6) | 2 (4.4) | - | - |
| Hemochromatosis (+ NASH/Alcohol) | 1 (1.8) | 1 (2.2) | 3 (4.8) | 1 (3.8) |
| Autoimmune hepatitis | - | - | - | - |
| Primary sclerosing cholangitis | - | - | - | 1 (3.8) |
| Other | - | - | 2 (3.2) | - |
| **Hepatitis status – no. (%)** | | | | |
| Hepatitis B or C positive | 17 (30.9) | 15 (33.3) | 16 (25.4) | 5 (19.2) |
| Chronic Hepatitis B | 10 (18.2) | 8 (17.8) | 11 (17.5) | 4 (15.4) |
| Chronic Hepatitis C | 8 (14.5) | 7 (15.6) | 5 (7.9) | 2 (7.7) |
| **Cirrhosis – no. (%)** | | | | |
| Yes | 22 (40) | 12 (26.7) | 24 (38.1) | 10 (38.5) |
| No | 33 (60) | 33 (73.3) | 39 (61.9) | 16 (61.5) |
| **Surgery – no. (%)** | | | | |
| (Extended) Hemi-hepatectomy | 30 (54.5) | 26 (57.8) | 28 (44.4) | 14 (53.8) |
| Partial resection (≥2 segments) | 12 (21.8) | 8 (17.8) | 24 (38.1) | 6 (23.1) |
| Partial resection (1 segment) | 11 (20.0) | 10 (22.2) | 11 (24.4) | 6 (23.1) |
| LTx | 2 (3.6) | 1 (2.2) | - | - |
| **Tumor differentiation – no. (%)** | | | | |
| Good | 8 (14.5) | 4 (8.9) | 8 (12.7) | 3 (11.5) |
| Moderate | 30 (54.5) | 22 (48.9) | 36 (57.1) | 15 (57.7) |
| Poor | 9 (16.4) | 9 (20) | 14 (22.2) | 5 (19.2) |
| Unknown | 8 (14.5) | 10 (22.2) | 5 (7.9) | 3 (11.5) |
| **Vascular invasion – no. (%)** | | | | |
| Yes | 23 (41.8) | 26 (57.8) | 41 (65.1) | 18 (69.2) |
| No | 27 (49.1) | 15 (33.3) | 22 (34.9) | 6 (23.1) |
| Unknown | 5 (9.1) | 4 (8.9) | - | 2 (7.7) |
| **BCLC stage – no. (%)** | | | | |
| 0 | 1 (1.8) | - | 4 (6.3) | - |
| A | 36 (65.5) | 30 (66.7) | 47 (74.6) | 22 (84.6) |
| B | 18 | 15 (33.3) | 12 (19.0) | 4 (15.4) |
| **Number of lesions – no. (%)** | | | | |

| | | | | |
|---|---|---|---|---|
| 1 | 30 (54.5) | 26 (57.8) | 49 (77.8) | 22 (84.6) |
| >1 | 25 (45.5) | 19 (42.2) | 14 (22.2) | 4 (15.4) |
| Median (range) | 1 (1-11) | 1 (1-10) | 1 (1-11) | 1 (1-11) |
| **Size of largest lesion (cm)** | | | | |
| Mean ± SD | 6.2 ± 4.3 | 9.1 ± 6.9 | 7.0 ± 4.7 | 8.0 ± 4.7 |
| Median (range) | 5.2 (1.3-24) | 7.5 (1-34) | 5.7 (0.8-21.0) | 7.15 (1.7-16.5) |
| **AFP level before resection (ug/l)** | | | | |
| Mean ± SD | 711 ± 2384 | 113360 ± 519877 | 1850 ± 6673 | 784 ± 1641 |
| Median (range) | 7 (1-10709) | 12 (1-3118700) | 9 (1-45803) | 47 (1-4973) |

**Table S2.** Primer sequences and annealing temperatures (Tm) used for RT-qPCR.

| Primer | Tm | Forward Primer | Reverse Primer | Product Length |
|---|---|---|---|---|
| **CAGE1** | 60 | TCATCCGAAGTCCATGACCA | GACTCTTCCTGGAGTGGTTG | 118 |
| **CBLL2** | 62 | TTCCACCAGAACAGCACACC | AACGGTTTCCCACTGGATGG | 146 |
| **CCDC83** | 60 | AGGAGGGCAGGCCTTTTTAATC | TCCATTGTGCTGGTTAGCTATGA | 148 |
| **CPXCR1** | 60 | CAGCCAGTCATACTATCCTC | CTACAGTCATTAGGAGGCTC | 118 |
| **CSAG2/3** | 58 | GGAGTGGGCCAACACTATCC | GGCTGTCCGAAGAGAGACTG | 123 |
| **CT45** | 62 | ATGCACATCACTCCCAGGTG | TTGTTTCCTTGCTGGAGGAGA | 147 |
| **CT47A1** | 60 | ACCTAGACGCAGCAGAGGT | AACTTGAACACTGTCACATACATCC | 141 |
| **CTAG1A/B** | 60 | GGCTTCAGGGCTGAATGGA | TGTTGCCGGACACAGTGAAC | 191 |
| **Cxorf48** | 60 | CTGGCAACGTGCCTCTAAAAG | AAGATGGCGAGGCACAACAT | 110 |
| **DDX53** | 60 | GTTGGTGTGGTCATTGGTTAC | CGCTTTGGCCTTTGCTTTCAT | 144 |
| **DPPA2** | 62 | CAATCTCCTTCCATCCCAGGGT | ACCAGTGTCAAAATCACACTTTCC | 118 |
| **DUSP21** | 62 | TTGTCAATGCCTCGGTGGAA | CGAGTCACGAGCATCGGTAA | 86 |
| **FAM46D** | 60 | AGCCTTAACGGATGAAGGAAAA | AAACTCCAGCTAGTGAAACTCC | 92 |
| **FATE1** | 62 | ATGGAGCTTGGATCTCGGTC | CTCAGCATTCTGGGCTTTGG | 155 |
| **FBXO39** | 60 | TGATAGATCTCCTGCCCACCT | CTCGTCGAGTGACTCATGGTT | 83 |
| **FMR1NB** | 60 | TCCTGCTGTTCGTGTGCTAC | TCAGCAAAGCTTCCAATGCG | 147 |
| **FTHL17** | 60 | ATCAACAGCCACATCACGCT | CATTTTGTCGTCCGACAGGC | 132 |
| **GAGE1** | 60 | ACCTGAG-<br>TCATCTTAAAACATGTGA | AGTAAACATGAAGCAGAGTGCC | 80 |
| **GPC3** | 60 | AACCATGTCTATGCCCAAAGGT | CCAGAGCCTCCAATGCACTC | 108 |
| **GUSB** | 58 | CAGGTGATGGAAGAAGTGG | GTTGCTCACAAGGTCACAG | 171 |
| **HORMAD1** | 60 | CAACGAATCTAGCATGTTGTC | CACAATCACCATCCTTAAAACC | 188 |
| **HPRT1** | 58 | GCTATAAATTCTTTGCTGAC-<br>CTGCTG | AATTACTTTTATGTCCCCTGTT-<br>GACTGG | 140 |
| **LUZP4** | 60 | CTTCGTTTCGGAAGCTAACGC | CTCCGATGGCGATGTCTATGA | 217 |
| **MAGEA1** | 60 | AGAAGCGAGGTTTCCATTCTGA | GGAATCCTGTCCTCTGGGTTG | 116 |
| **MAGEA2** | 62 | CTCCAGCTTCTCGACTACCATC | GACTCCAGGTCGGGAAACATTC | 148 |
| **MAGEA3** | 62 | ATCTTCAGCAAAGCTTCCAGT | GGTGGCAAAGATGTACAAGTGG | 93 |
| **MAGEA4** | 58 | GAGCTTCTGCGTCTGACTCG | TGTCTGCTCAGAACCTTGTCTC | 85 |
| **MAGEA8** | 60 | GGTCGGCTTGAGATCGGCT | CCTCAGCTTGACTGCTACTACTG | 150 |
| **MAGEA9B** | 60 | GCTTGATACCGGTGGAGGAG | GGTTAGCCTGTCCCGAGAAC | 124 |
| **MAGEA10** | 62 | GAGATCGGCTGAAGAGAGCG | ACTCTTGTCAGATCCTGCGAC | 140 |
| **MAGEB1** | 60 | TGAAGTAGTGAGCAGCCAAGA | GCTGGCAGCACCAATAAATGT | 172 |
| **MAGEB2** | 58 | TCCTGACTTCCGCTTTGGAGGC | GCACGGAGCTTACTCTTCTGACC | 135 |

| | | | | |
|---|---|---|---|---|
| **MAGEB3** | 60 | CTACCCAAACCTCTTCTCAGCC | AGACCCTGGATCCTCCCTCTA | 144 |
| **MAGEB6** | 62 | ACCCTTGTCAGCAAGCTAGG | GATCACAACCAGGAGCGACA | 99 |
| **MAGEC1** | 62 | GGCCATCTTGGGAGTCTGAA | TGGAGCACCTTGAAGACTGG | 106 |
| **MAGEC2** | 62 | GGAGTCAAGGCCTGTTGGAT | GGGAGGCATGACGACTTCTT | 84 |
| **PAGE1** | 62 | GGCTGAAGTTGTGAAATATGGGT | CTGCAGATGCTCCCTCATCC | 177 |
| **PAGE5** | 62 | TGATGTCAGGGAGGGGACTC | TGGTTTCAGTCTTCATTTGTCTTGG | 105 |
| **PASD1** | 62 | TGCAGAGGTTGAGCAGTATGG | GGATTCACCTCAGGCTCACC | 153 |
| **PLAC1** | 60 | ACACAGCAAGTTCCTTCTTCC | GAGGATTTCTTCTTCTGGCAGC | 118 |
| **PMM1** | 58 | CGAGTTCTCCGAACTGGAC | CTGTTTTCAGGGCTTCCAC | 86 |
| **RNF17** | 60 | GGACAATGCAGTGGTCCAAAG | AGGAGCACCAAGAGAATCGAA | 137 |
| **SAGE1** | 58 | CCTTAGCTGACTCTGGTGCTC | GACTCGTTTGAAGTGGAGAAGC | 150 |
| **SLCO6A1** | 62 | TGGCCTTGGGTGTAAGCTATG | ATCCAACAACGTCCTGTGTG | 136 |
| **SPANXA** | 62 | ATGATGCCGGAGACCCCAAC | GTGGTCATTCAGCAGTTCCTCT | 144 |
| **SPANXC** | 60 | CGCTACAGGAGGAACGTGAA | ATTCCTCCTCCTCCATTTGG | 100 |
| **SPANXN3** | 62 | ACCAGAATCATGGAACAGCCAA | TGTTTGGTACCTCTTGCATCTC | 106 |
| **SYCP1** | 62 | CTATCTGTGGACATCTGCCAA | TTGGTTTTGTTGGTGTCTTCAC | 80 |
| **TEKT5** | 62 | GGTCCATGACAACGTGGAGA | TGCTGAGCATCCCGGTTATC | 126 |
| **TFDP3** | 60 | TTGGAGGTGTGTTCACGACG | CTGAGATCCACCGGAGCTTG | 113 |
| **TPPP2** | 60 | GCAAAGTCAAGGCCAAGAACG | CTGGACTCTTCCCTTTGAAGC | 99 |
| **TSPY** | 62 | ACAAGATTGCTGAGTCCCCTG | TCAACAACTGGGAGTCCCCT | 149 |
| **ZCCHC13** | 62 | TGCTACAACTGTGGGAGAAGC | TGACGATCACAGTCACGAGC | 122 |

**Table S3.** Results of the literature search and overview of included articles.

| Study | Gene(s) | Population | Detection method | Outcome |
|---|---|---|---|---|
| **Wei Y, et al. Int J Oncol. 2018 [2]** | MAGEA9 | HCC patients (n=90; China) | IHC | IHC: 40/90 (44%) MAGEA9+ |
| **Jiao Y, et al. PLoS One. 2017 [3]** | TFDP3 | HCC cell line (HepG2) and normal human hepatocyte cell line (L-02) and HCC patients (China) | RT-qPCR and IHC | mRNA and IHC: HepG2 and L02 are both TFDP3+, expression is higher in HepG2. Also protein expression in HCC patients. |
| **Liu, et al. Cancer Lett. 2017 [4]** | CTCFL | HCC cell lines (HepG2, SMMC-7721, Huh7, HCCLM3, PLC/PRF/5), normal human hepatocyte cell lines (L-02 and WRL68) and HCC patients (n=25; China) | RT-qPCR and IHC | RT-qPCR: all cell lines positive, expression higher in HCC cell lines than normal human hepatocyte cell lines<br>IHC: 18/25 (72%) $CTFCL^{high}$ and 7/25 (28%) $CTCFL^{low}$ |
| **Xie, et al. Drug target. 2017 [5]** | TTK | Review | n.a. | Liu, Oncotarget 2015: 118/152 (77.63%) of HCC patients mRNA TTK+ – China |
| **Charoentong, et al. Cell Rep. 2017 [6]** | BRDT, CAGE1, CCDC83, CPXCR1, CSAG2, CT45A1, DDX53, DPPA2, | The Cancer Genome Atlas (TCGA); including 363 HCC patients | RNA sequencing | Aforementioned genes are all correlated with CD4 and/or CD8 T cells in HCC |

| | | | | |
|---|---|---|---|---|
| | FMR1NB, FTHL17, GAGE1, LUZP4, MAGEA1, MAGEA2, MAGEA3, MAGEA4, MAGEA5, MAGEA9, FAM46D, MAGEB1, MAGEB2, MAGEB3, MAGEC1, PAGE1, PASD1, POTEA, POTEB, POTED, SLCO6A1, SPANXC, SPANXN3, SSX3, SSX5, SSX7, TSPY2, TSPY3, TSSK6, XAGE2, ZNF645, | | | |
| **Kido, et al. J Genet Genomics. 2016 [7]** | TSPY | TCGA and refers to Kido, et al. 2014<br>IHC: male HCC patients (n=287; TMA purchased from US Biomax) and RT-qPCR: male HCC patients (n=32; China) | TCGA: RNA seq<br>Kido, et al. 2014: RT-qPCR and IHC | This paper researches the TSPY co-expression network (TCN), which is activated in 30% of HCCs (TCGA).<br>Kido, et al. 2014: RT-qPCR: 15/32 (46.9%) TSPY+<br>IHC: 55/287 (19.2%) TSPY+ |
| **Fu, et al. Int J Clin Exp Pathol. 2015 [8]** | ACRBP | HCC cell lines (Bel-7404[1], HepG2[2], QGY-7703[3], QGY-7701[4], BEL-7402[5], SMMC-7721[6]) | RT-PCR[1-6], IHC[1,2] and WB[1,2] | RT-PCR: 6/6 cell lines<br>IHC: 2/2 cell lines<br>WB: 2/2 cell lines |
| **Wang, et al. Int J Clin Exp Pathol. 2015 [9]** | MAGEA3, MAGEA4, MAGEC2, NY-ESO-1 | HCC cell lines (LO2, HepG2, Hep3B, Huh7, SMMC-7721) and HCC patients (China; n=142) | RT-PCR and IHC | Cell lines: 4/5 cell lines for 4 TAAs.<br>HCC patients: 112/142 (78.9%) MA3+, 48/142 (33.8%) MA4+, 106/142 (74.6%) MC2+, 20/142 (14.1%) NY-ESO-1+. No expression in TFL.<br>IHC: 108/142 (76.1%) MA3+, 44/142 (31.0%) MA4+, 99/142 (69.7%) MC2+, 19/142 (13.4%) NY-ESO-1+ |
| **Sideras, et al. Br J Cancer. 2015 [10]** | MAGEA1, MAGEA3/4, MAGEA10, MAGEC1, MAGEC2, NY-ESO-1, SSX2, SP17 | HCC patients (Netherlands; n=133) | IHC | 9.8% MAGEA1+, 3.0% MAGEA3/4+, 7.5% MAGEA10+, 17.3% MAGEC1+, 19.5% MAGEC2+, 3.8% NY-ESO-1+, 0% SSX2+, 87% SP17+. No expression in TFL, except SP17 (88.0%) |
| **Melis, et al. J Transl Med. 2014 [11]** | NUF2, TTK, MAGEA3, CEP55 | HBV+ HCC patients (n=10; Italy) | RT-PCR | Expression of al 4 TAAs in 10 patients, both in HCC and TFL, but higher in HCC. |
| **Li, et al. J Transl Med. 2014 [12]** | TSPY | HCC cell lines (HepG2, SMMC7721, Huh7, MHCC97L, MHCC97H, HCCLM3) and HCC patients (n= 52;China) | RT-PCR | 6/6 cell lines and expression of TSPY in male HCC tissues, but not female HCC tissues |

| | | | | |
|---|---|---|---|---|
| **Deng, et al. Hepatology. 2014 [13]** | DUSP21, CT45, ZCCHC13, MAGEA9, MAGEB6, PIHD3, PNMA5, MPC1L, IL13RA1 | HCC patients (n=24; China?) | RT-PCR | 8/24 (33.3%) DUSP21+, 7/24 (29.2%) CT45+, 4/24 (16.7%) ZCCHC13+, 3/24 (12.5%) MAGEA9+, 3/24 (12.5%) MAGEB6+, 4/24 (16.7%) PIHD3+, 6/24 (25%) PNMA5+, 6/24 (25%) MPC1L+, 1/24 (4.2%) IL13RA1+ |
| **Xia, et al. Int J Clin Exp Pathol. 2013 [14]** | SP17, MAGEC1, NY-ESO-1 | HCC patients (n=45; China) | IHC | 16/45 (35.6%) MAGEC1+, 7/45 (15.6%) NY-ESO-1+, 36/45 (80%) SP17+ |
| **Zhou, et al. Oncol Rep. 2013 [15]** | FAM9C | HCC cell lines (SSMC-7721, QGY-7703, BEL-7404, BEL-7405, YY-8103, Huh7) and HCC patients (n=46; China) | RT-qPCR and IHC | RT-qPCR: 25/46 HCC patients have upregulation of FAM9C in T compared to TFL<br>Cell lines: 2/6 FAM9C+<br>IHC showed nuclear staining (T>TFL) |
| **Chen, et al. Genet Test Mol Biomarkers. 2013 [16]** | CTCFL | HCC cel lines (SMMC-7721, BEL-7402, Huh7, HepG2) and HCC patients (n=105; China) | RT-PCR, IHC and WB | Cell lines: 3/4 CTCFL+ (RT-PCR and WB)<br>HCC patients: 58/105 (55.2%) CTCFL+ (IHC) |
| **Song, et al. Oncol Rep. 2012 [17]** | AKAP3, CTp11 | HCC cell lines (SNU-354, SNU-398, SNU-423, SNU-449, HepG2) and HCC patients (n= 10; Korea) | RT-PCR | 5/10 (50%) AKAP3+, 1/9 (11.1%) CTp11+ |
| **Li, et al. Bull Cancer. 2012 [18] – no full text** | CABYR-c | HCC patients (n=20; China) | RT-PCR and WB | Both mRNA and protein expression are significantly higher in HCC compared to TFL |
| **Yoon, et al. Tohoku J Exp Med. 2011 [19]** | RNF17 | HCC patients (n=28; Korea), CCA patients (n=5) and combined HCC-CCA (n=8) – Korea | RT-qPCR | 4/28 (14.3%) HCC RNF17+, 1/5 (20%) CCA RNF17+, 2/8 (25%) combined HCC/CCA RNF17+. No expression in TFL. |
| **Tseng, et al. Oncol Rep. 2011 [20]** | CABYR-a/b, CABYR-c/d, CABYR-e | HCC cell lines (HepG2, Huh7) and HCC patients (n=16; Taiwan) | RT-PCR and WB | Cell lines: 2/2 expressed CABYR-a/b and CABYR-c/d<br>HCC patients: 7/16 (43.8%) CABYR-a/b+, 14/16 (87.5%) CABYR-c/d+, 0/16 (0%) CABYR-e+ |
| **Wang, et al. Oncol Rep. 2009 [21]** | NY-ESO-1, CTAG2, SSX1 | HCC patients (n=64; China) | RT-PCR | 19/64 (29.7%) NY-ESO-1+, 29/64 (45.3%) CTAG2+, 24/64 (37.5%) SSX1+ |
| **Riener, et al. Int J Cancer. 2009 [22]** | MAGEA4, MAGEC1, MAGEC2, GAGE, NY-ESO-1 | HCC patients (n=146; Switzerland), CCA (n=50), GBC (n=32) | IHC | HCC: 0/146 (0%) MAGEA4+, 17/146 (12%) MAGEC1+, 50/146 (34%) MAGEC2+, 16/146 (11%) GAGE+, 3/146 (2%) NY-ESO-1+. No expression in CCA.<br>GBC: 4/32 (13%) MAGEC2+, 1/32 (3%) GAGE+, 1 (3%) NY-ESO-1+, 0/32 MAGEC1/MAGEA4+ |
| **Lu, et al. Chin Med J. 2007 [23]** | NY-ESO-1, SSX1 | HCC patients (n=36; China) | RT-PCR | 4/36 (11.1%) NY-ESO-1+, 22/36 (61.1%) SSX1+ |

| | | | | |
|---|---|---|---|---|
| **Wu, et al. Life Sci. 2006 [24]** | SSX2, SSX5 | HCC patients (n=36; China) | RT-PCR | 13/36 (36.1%) SSX2, 17/36 (47.2%) SSX5 |
| **Watanabe, et al. Cancer Sci. 2005 [25]** | IGSF11 | HCC cell line (Alexander, Huh7, HepG2, SNU475) | RT-PCR | HCC cell lines: 3/4 IGSF11+ |
| **Yin, et al. Br J Cancer. 2005 [26]** | TSPY | HCC cell lines (hep-hcc-1, hep-hcc-2, hep-hcc-HLE, Hep3B, COS7) and HCC patients (n= 57;China) | RT-PCR | 20/57 (35%) TSPY+ |
| **Shi, et al. Br J Cancer. 2005 [27]** | DDX53 | HCC patients (n=33; China) | RT-PCR | 13/33 (39.4%) DDX53+ |
| **Peng, et al. Cancer Lett. 2005 [28]** | MAGEA1, MAGEA3, MAGEA4, MAGEA10, SSX1, SSX2, SSX4, SSX5, NY-ESO-1, MAGEB1, MAGEB2, MAGEC1, MAGEC2, SYCP1 | HCC patients (n=73; China) | RT-PCR | 51/73 (69.9%) MAGEA1+, 35/73 (47.9%) MAGEA3+, 6/30 (20%) MAGEA4+, 11/30 (36.7%) MAGEA10+, 29/43 (67.4%) SSX1+, 26/73 (35.6%) SSX2+, 21/43 (48.8%) SSX4+, 13/43 (30.2%) SSX5+, 31/73 (42.5%) NY-ESO-1+, 13/25 (52%) MAGEB1+, 15/25 (60%) MAGEB2+, 12/25 (48%) MAGEC1+, 17/25 (68%) MAGEC2+, 10/30 (33.3%) SYCP1+ |
| **Sato, et al. Int J Oncol. 2005 [29] – no full text** | NY-ESO-1, CTAG2 | HCC patients – Japan | RT-PCR and IHC | IHC: 3/10 (30%) NY-ESO-1+ - all 10 samples expressed NY-ESO-1 mRNA<br>1/6 (16.7%) CTAG2+ - all 6 samples expressed CTAG2 mRNA |
| **Yang, et al. Lab Invest. 2005 [30]** | FATE | HCC patients (n=35; China) | RT-PCR and IHC | RT-PCR: 10/15 (66%) FATE+<br>IHC: 7/35 (20%) FATE+ |
| **Dong, et al. Biochem Cell Biol. 2004 [31] – no full text** | FATE | HCC patients (China) | RT-PCR | 25% of HCC samples FATE+ |
| **Dong, et al. Br J Cancer. 2004 [32]** | ZNF165 | HCC patients (n=42; China) | RT-PCR | 22/42 (52%) ZNF165+ |
| **Zhao, et al. World J Gastroenterol. 2004 [33]** | MAGEA1, MAGEC2, SSX1, SPANXC | HCC patients (n=105; China) | RT-PCR | 79/105 (75.2%) MAGEA1+, 59/105 (56.2%) MAGEC2+, 76/105 (72.4%) SSX1+, 66/105 (62.9%) SPANXC+ |
| **Li, et al. Lab Invest. 2003 [34] – no full text** | MAGEC2 | HCC patients (n=70; China) | IHC | 26/70 (37.1%) MAGEC2+ |
| **Dong, et al. Br J Cancer. 2003 [35]** | FATE, TPTE | HCC patients (n=62; China) | RT-PCR | 41/62 (66%) FATE1+, 24/62 (39%) TPTE+ |
| **Luo, et al. Cancer Immun. 2002 [36]** | MAGEA1, MAGEA3, MAGEA4, GAGE, NY-ESO-1, SSX1, SSX2, SSX4, SYCP1, LUZP4 | HCC patients (n=21; China) | RT-PCR | 4/21 (19%) MAGEA1+, 5/21 (24%) MAGEA3+, 1/21 (4.8%) MAGEA4+, 8/21 (38%) GAGE+, 0/21 (0%) NY-ESO-1+, 8/21 (38%) SSX1+, 2/21 (9.5%) SSX2+, 2/21 |

| | | | | |
|---|---|---|---|---|
| | | | | (9.5%) SSX4+, 6/21 (29%) SYCP1+, 4/21 (19%) LUZP4+ |
| **Wang, et al. J Immunol. 2002 [37]** | MAGEC2, TFDP3 | HCC patients (n=20; China) | RT-PCR | 14/20 (70%) MAGEC2+, 5/17 (29.4%) TFDP3+ |
| **de Wit, et al. Int J Cancer. 2002 [38]** | DSCR8 | HCC cell lines (Hep3B, HepG2, PLC/RPF/5, Huh7) | RT-PCR | 1/4 cell lines DSCR8+ |
| **Ono, et al. Proc Natl Acad Sci U S A. 2001 [39]** | ACRBP | HCC patients (n=5; Japan) | RT-PCR | 2/5 (40%) ACRBP+ |
| **Chen, et al. Cancer Lett. 2001 [40]** | SSX1, SSX2, SSX4, SSX5, SYCP1, NY-ESO-1 | HCC patients (n=30; Taiwan) | RT-PCR | 24/30 (80%) SSX1+, 14/30 (46.7%) SSX2+, 22/30 (73.3%) SSX4+, 10/30 (33.3%) SSX5+, 2/30 (6.7%) SYCP1+, 11/30 (36.7%) NY-ESO-1+ |

**Table S4.** Frequency table of healthy liver tissues (n=21) expressing mRNA of the CTAs. Colors correlate to the percentage of positive healthy liver tissues.

| | **mRNA$^+$ healthy liver (%)** | | **mRNA$^+$ healthy liver (%)** |
|---|---|---|---|
| **CAGE1** | 0.0 | **MAGEA10** | 0.0 |
| **CBLL2** | 42.9 | **MAGEB1** | 19.0 |
| **CCDC83** | 0.0 | **MAGEB2** | 0.0 |
| **CPXCR1** | 0.0 | **MAGEB3** | 28.6 |
| **CSAG2/3** | 85.7 | **MAGEB6** | 28.6 |
| **CT45** | 14.3 | **MAGEC1** | 4.8 |
| **CT47A1** | 0.0 | **MAGEC2** | 0.0 |
| **Cxorf48** | 0.0 | **NYESO1** | 0.0 |
| **DDX53** | 47.6 | **PAGE1** | 0.0 |
| **DPPA2** | 0.0 | **PAGE5** | 100.0 |
| **DUSP21** | 23.8 | **PASD1** | 0.0 |
| **FAM46D** | 0.0 | **PLAC1** | 0.0 |
| **FATE1** | 33.3 | **RNF17** | 4.8 |
| **FBXO39** | 100.0 | **SAGE1** | 0.0 |
| **FMR1NB** | 0.0 | **SLCO6A1** | 0.0 |
| **FTHL17** | 42.9 | **SPANXA** | 66.7 |
| **GAGE1** | 23.8 | **SPANXC** | 38.1 |
| **HORMAD1** | 100.0 | **SPANXN3** | 0.0 |
| **LUZP4** | 0.0 | **SYCP1** | 47.6 |
| **MAGEA1** | 0.0 | **TEKT5** | 100.0 |
| **MAGEA2** | 23.8 | **TFDP3** | 33.3 |
| **MAGEA3** | 14.3 | **TPPP2** | 100.0 |
| **MAGEA4** | 0.0 | **TSPY** | 0.0 |
| **MAGEA8** | 0.0 | **ZCCHC13** | 38.1 |
| **MAGEA9** | 0.0 | | |

**Table S5.** Expression of excluded CTAs in HCC patients and in cirrhotic liver tissues without malignancy.

| | mRNA-positive HCC (%)[1] | mean in mRNA-+ HCC (range)[2] | Relative expression HCC (compared to testis)[3] | mRNA-positive TFL (%)[4] | mean in mRNA-+ TFL (range)[5] | Relative expression TFL (compared to testis)[6] | mRNA-positive cirrhotic tissue[7] |
|---|---|---|---|---|---|---|---|
| **CCDC83** | 0.00 | | | 0.00 | | | 0 |
| **CPXCR1** | 2.02 | 0.001 (0.001-0.001) | 0.00378 | 0.00 | | | 0 |
| **Cxorf48** | 8.25 | 0.157 (0.002-0.991) | 1.839 | 2.04 | 0.012 (0.001-0.023) | 0.139 | 2.9 |
| **DPPA2** | 1.03 | 0.135 (0.135-0.135) | 1.204 | 0.00 | | | 0 |
| **FAM46D** | 5.15 | 0.003 (0.002-0.004) | 0.058 | 0.00 | | | 2.9 |
| **FMR1NB** | 6.19 | 0.031 (0.002-0.124) | 0.022 | 1.02 | 0.088 (0.088-0.088) | 0.062 | 0 |
| **LUZP4** | 6.19 | 0.106 (0.001-0.49) | 0.242 | 1.02 | 0.279 (0.279-0.279) | 0.636 | 0 |
| **MAGEA4** | 6.19 | 0.803 (0.001-2.559) | 28.036 | 0.00 | | | 0 |
| **MAGEA8** | 3.09 | 0.014 (0.004-0.022) | 5.115 | 0.00 | | | 0 |
| **PASD1** | 2.02 | 0.017 (0.007-0.026) | 0.007 | 2.00 | 0.02 (0.019-0.021) | 0.009 | 0 |
| **PLAC1** | 4.12 | 0.014 (0.001-0.041) | 0.146 | 0.00 | | | 0 |
| **RNF17** | 21.65 | 0.053 (0.001-0.507) | 0.04570 | 13.27 | 0.023 (0.002-0.134) | 0.01964 | 5.7 |
| **SAGE1** | 4.12 | 0.086 (0.008-0.15) | 0.505 | 3.06 | 0.19 (0.006-0.543) | 1.117 | 0 |
| **SPANXN3** | 1.01 | 0.004 (0.004-0.004) | 52.644 | 3.00 | 0.002 (0.001-0.003) | 27.174 | 0.0 |

[1]Percentage of hepatocellular carcinomas (HCC) expressing mRNA of the excluded CTAs – meaning a Ct-value <35 and relative expression > 0.001 (n=100); [2]Mean relative expression (relative to the geometric mean of the 3 household genes- GUSB, HPRT1, PMM1) level in HCCs expressing the CTA and range; [3]Mean relative expression of the CTA in HCC expressing the CTA, relative to the relative mean expression in 3 testis tissues; [4]Percentage of paired tumor-free liver tissues (TFL) expressing mRNA of the excluded CTAs (n=100); [5]Mean relative expression level in TFLs expressing the CTA and range; [6]Mean relative expression of the CTA in TFL expressing the CTA, relative to the relative mean expression in 3 testis tissues; [7]Percentage of non-cancerous cirrhotic liver tissues expressing the CTA (n=35)

**Table S6.** Patient characteristics of HCC-patients included in protein expression analysis.

| Characteristic | HCC patients (n=76) |
|---|---|
| **Age at surgery (years)** | |
| Mean ± SD | 60.4 ± 14.4 |
| Median (range) | 64 (16-82) |
| **Sex – no. (%)** | |
| Male | 47 (61.8) |
| Female | 29 (38.2) |
| **Race – no. (%)** | |
| White | 64 (84.2) |
| African | 6 (7.9) |
| Asian | 5 (6.6) |
| Not reported | 1 (1.3) |
| **Etiology – no. (%)** | |
| No known liver disease | 21 (27.6) |
| Alcohol | 17 (22.4) |
| Hepatitis B | 9 (11.8) |
| NASH | 8 (10.5) |
| Hepatitis C + Alcohol | 6 (7.9) |
| Hepatitis B + Alc/HepC/HepD/NASH | 6 (7.9) |
| Hepatitis C | 5 (6.6) |
| Fibrolamellar HCC | 3 (4.0) |

| | |
|---|---|
| Hemochromatosis + NASH | 1 (1.3) |
| Autoimmune hepatitis | - |
| Primary sclerosing cholangitis | - |
| **Hepatitis status – no. (%)** | |
| Hepatitis B or C positive | 26 (34.2) |
| Chronic Hepatitis B | 15 (19.7) |
| Chronic Hepatitis C | 12 (15.8) |
| **Cirrhosis – no. (%)** | |
| Yes | 23 (30.3) |
| No | 53 (69.7) |
| **Tumor differentiation – no. (%)** | |
| Good | 8 (10.5) |
| Moderate | 41 (54.0) |
| Poor | 14 (18.4) |
| Unknown | 13 (17.1) |
| **Vascular invasion – no. (%)** | |
| Yes | 40 (52.6) |
| No | 29 (38.2) |
| Unknown | 7 (9.2) |
| **Number of lesions – no. (%)** | |
| 1 | 40 (52.6) |
| >1 | 36 (47.4) |
| Median (range) | 1 (1-11) |
| **Size of largest lesion (cm)** | |
| Mean ± SD | 7.4 ± 5.2 |
| Median (range) | 6.1 (1-24) |
| **AFP level before resection (ug/l)** | |
| Mean ± SD | 64965 ± 401956 |
| Median (range) | 9.5 (2-3118700) |

**Table S7.** Cox regression analysis of HCC recurrence and HCC-specific survival based on CTA protein expression in TFL.

| | **Early recurrence** | | | | **HCC-specific survival** | | | |
|---|---|---|---|---|---|---|---|---|
| | Univariate analysis | | Multivariate analysis | | Univariate analysis | | Multivariate analysis | |
| **Variable** | HR (95% CI) | p-value | HR (95% CI) | p-value | HR (95% CI) | p-value | HR (95% CI) | p-value |
| **≥1 CTA in TFL** | 1.9 (0.9-3.9) | 0.092 | 2.5 (1.2-5.2) | 0.02 | 2.6 (1.1-6.1) | 0.03 | 3.8 (1.5-9.6) | 0.004 |
| **Number of CTAs in TFL (numeric)** | 1.9 (0.98-3.7) | 0.058 | | | 2.7 (1.2-6) | 0.012 | | |
| **>2 tumors** | 4.7 (2-11) | 0.00029 | 3.7 (1.5-8.9) | 0.004 | 2.6 (0.9-7.2) | 0.066 | | |
| **Chronic viral hepatitis** | 3.4 (1.6-7.1) | 0.0011 | 2.8 (1.3-6.2) | 0.01 | 3.1 (1.3-7.3) | 0.01 | 4.4 (1.8-11.1) | 0.001 |
| **Vascular invasion** | 1.6 (0.73-3.7) | 0.23 | | | 1.3 (0.5-3.1) | 0.57 | | |
| **Tumor > 5 cm** | 1.1 (0.49-2.3) | 0.89 | | | 1.7 (0.7-4.5) | 0.26 | | |
| **AFP > 400 ug/l** | 1.9 (0.84-4.3) | 0.12 | | | 2.2 (0.9-5.6) | 0.083 | | |

Abbreviations: HR, hazard ratio; CI, confidence interval; CTA, cancer-testis antigen; TFL, tumor-free liver; AFP , alphafetoprotein

**Table S8.** Cox regression analysis of HCC recurrence and HCC-specific survival based on CTA mRNA expression in HCC tumors.

| | **HCC recurrence** | | **HCC survival** | |
|---|---|---|---|---|
| | Univariate analysis | | Univariate analysis | |
| **Variable** | HR (95% CI) | p-value | HR (95% CI) | p-value |
| **≥1 CTA in tumor** | 1.8 (0.86-3.6) | 0.12 | 1 (0.41-2.6) | 0.94 |
| **≥2 CTAs in tumor** | 1.1 (0.65-1.9) | 0.67 | 0.86 (0.39-1.9) | 0.7 |
| **≥3 CTAs in tumor** | 1.1 (0.62-1.8) | 0.85 | 0.74 (0.34-1.6) | 0.47 |
| **Number of CTAs in tumor (numeric)** | 1 (0.96-1.1) | 0.29 | 1 (0.87-1.1) | 1 |
| **>1 tumor** | 1.2 (0.68-2) | 0.56 | 1.1 (0.49-2.4) | 0.83 |
| **>2 tumors** | 2.6 (1.3-4.9) | **0.0042** | 1.8 (0.69-4.9) | 0.22 |
| **Cirrhosis** | 1.6 (0.89-2.8) | 0.12 | 1.5 (0.66-3.4) | 0.33 |
| **Chronic viral hepatitis** | 2.3 (1.3-4) | **0.0031** | 3.3 (1.5-7.2) | **0.0032** |
| **Vascular invasion** | 1.3 (0.72-2.3) | 0.41 | 2.2 (0.96-4.9) | 0.063 |
| **Tumor > 5 cm** | 1.3 (0.74-2.3) | 0.37 | 2.3 (0.9-5.7) | 0.081 |
| **AFP > 200 ug/l** | 1.9 (1-3.4) | **0.034** | 2.7 (1.2-6) | **0.013** |
| **AFP > 400 ug/l** | 2.4 (1.3-4.5) | **0.0051** | 3.3 (1.5-7.3) | **0.0038** |

Abbreviations: HR, hazard ratio; CI, confidence interval; CTA, cancer-testis antigen; AFP, alphafetoprotein

**Table S9.** Antibodies used for immunohistochemistry.

| **Antibody** | **Host Species** | **Dilution** | **Company** | **Clone** | **Lot number** | **Procedure** | **Ab incubation at 37°C** |
|---|---|---|---|---|---|---|---|
| PAGE1 | Rabbit | 1:1000 | Sigma-Aldrich | Polyclonal | R04065 | Optiview CC1 32' | 32 minutes |
| TSPY | Rabbit | 1:200 | Sigma-Aldrich | Polyclonal | R59337 | Optiview CC1 32' | 32 minutes |
| MAGEA9 | Mouse | 1:50 | Prof. Y. Fradet, Québec, Canada [41] | 14A11 | N/A | Optiview CC1 32' | 32 minutes |
| MAGEC2 | Rabbit | 1:500 | Sigma-Aldrich | Polyclonal | A115364 | Optiview CC1 32' | 32 minutes |
| CT47A1 | Rabbit | 1:8000 | Sigma-Aldrich | Polyclonal | R39285 | Optiview CC1 32' | 32 minutes |
| MAGEA1 | Mouse | 1:50 | Santa Cruz | MA454 | B0507 | Optiview CC1 32' | 32 minutes |
| MAGEB2 | Rabbit | 1:500 | Sigma-Aldrich | Polyclonal | R109336 | Optiview CC1 32' | 32 minutes |
| SLCO6A1 | Rabbit | 1:200 | Sigma-Aldrich | Polyclonal | R72094 | Optiview CC1 32' | 32 minutes |
| MAGEC1 | Mouse | 1:3200 | Santa Cruz | CT7-33 | A1807 | Optiview CC1 32' | 32 minutes |

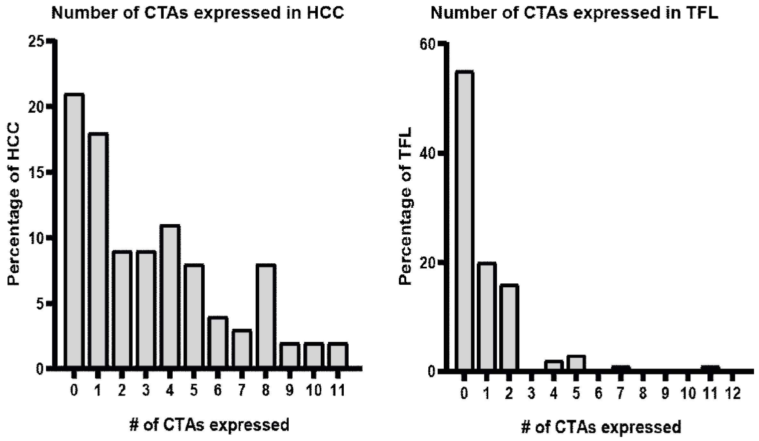


**Figure S1.** Number of CTAs co-expressed in HCC tumors and TFL, based on mRNA expression.

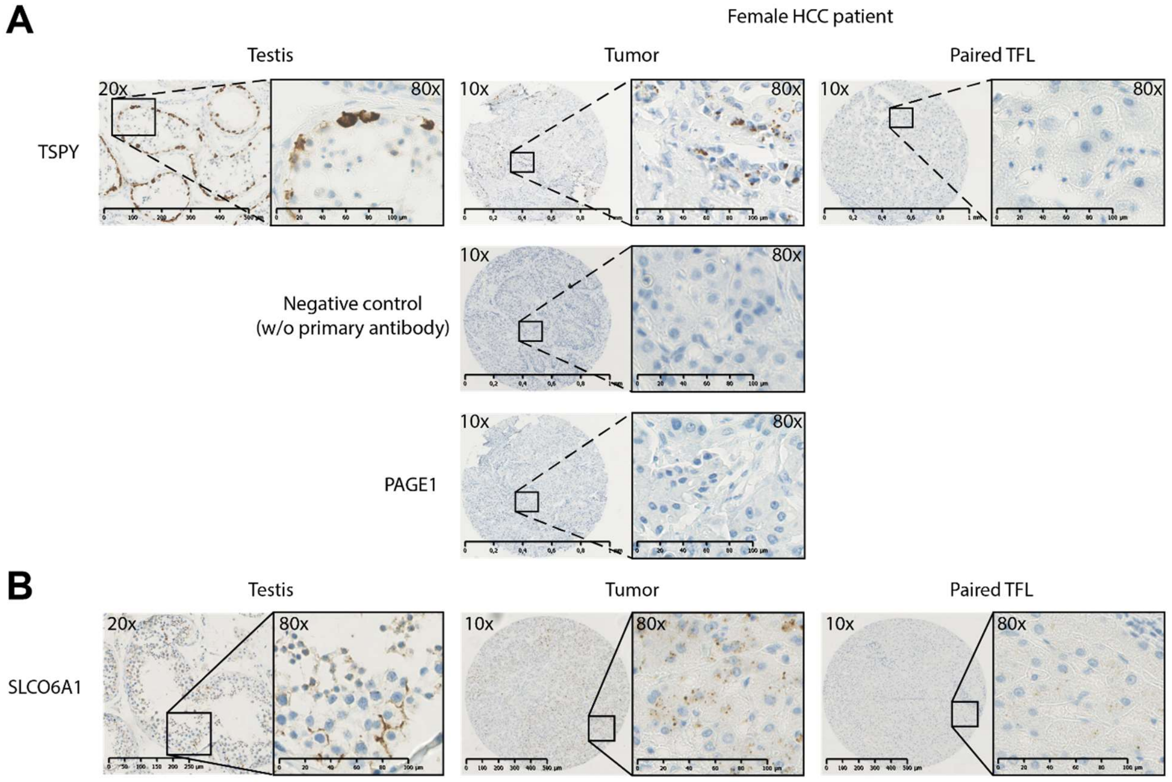


**Figure S2.** TSPY expression in female HCC tumors and SLCO6A1 expression. **A.** TSPY protein expression was determined by IHC. TSPY is expressed in spermatogonia of normal testis, as expected [42]. However, TSPY protein expression was also found in two female HCC patients, of which one example is shown above. The staining is absent in the negative control and in the PAGE1 stained core. TSPY is encoded by the y-chromosome, expression in women is thus biologically impossible. **B.** Representative example of immunohistochemical stains of SLCO6A1 in testis, a positive HCC tumor tissue and the paired TFL tissue.

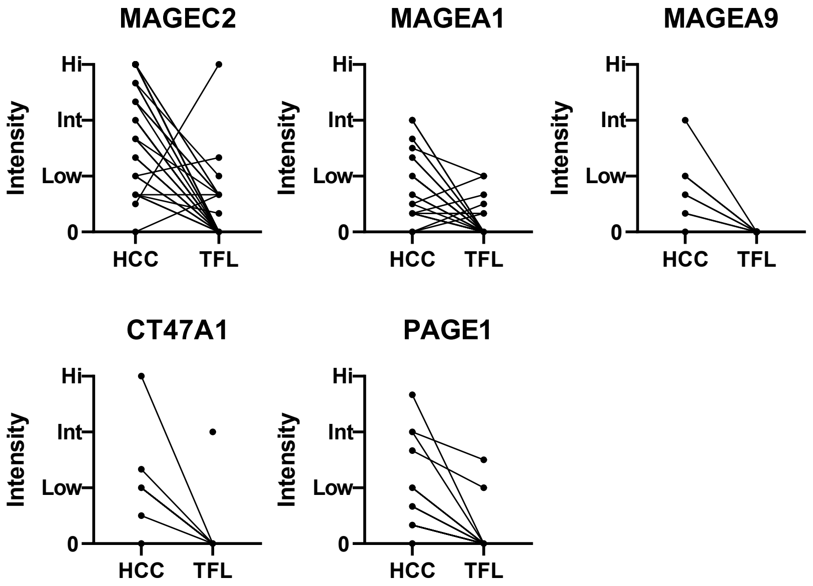


**Figure S3.** Protein expression of CTAs in HCC tumors paired tumor free liver. TMAs of tumor and TFL tissues were immunohistochemically stained to study the protein expression of aforementioned CTAs. The average intensity scores of three different tissue cores is depicted.

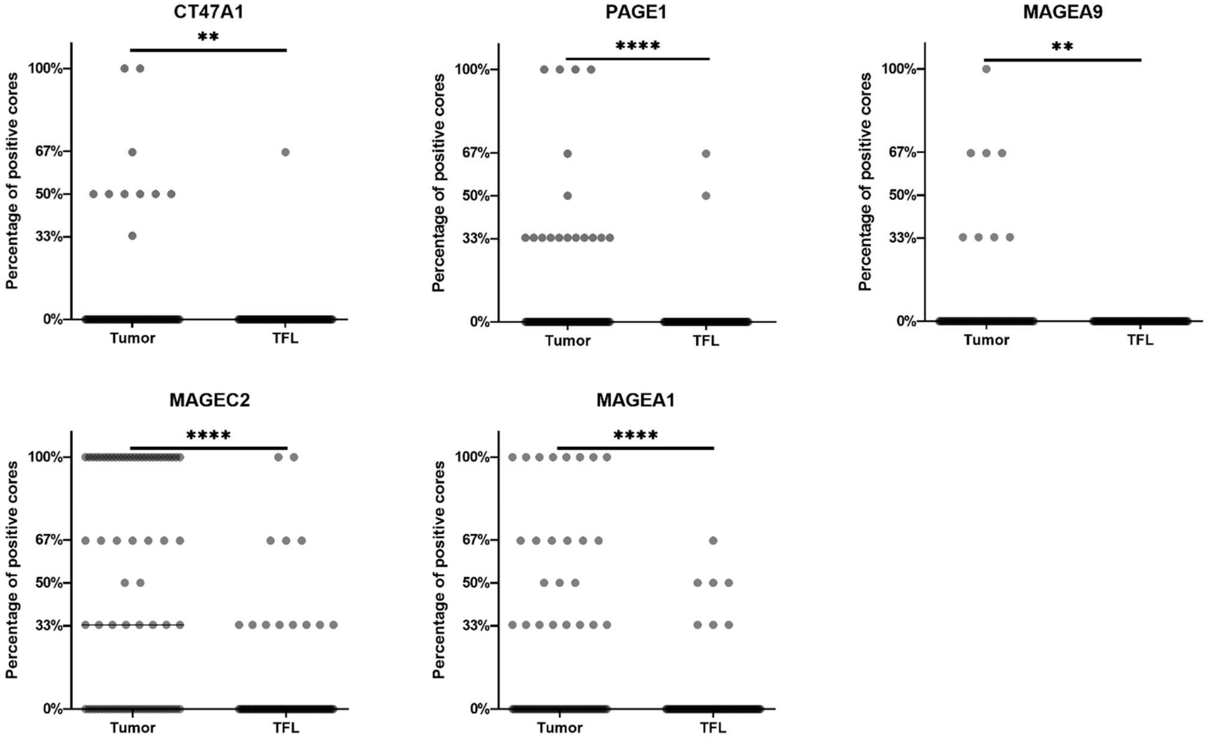


**Figure S4.** Proteins are focally expressed in most tumors. Protein expression was determined on TMAs, which had 3 cores of each tumor and TFL. The graphs display the percentage of cores containing protein-expressing cells (a score ≥1A). Most tumors and TFL express the proteins focally, illustrated by not all cores being positive. Wilcoxon signed-rank test. ** P<0.01, **** P<0.0001.

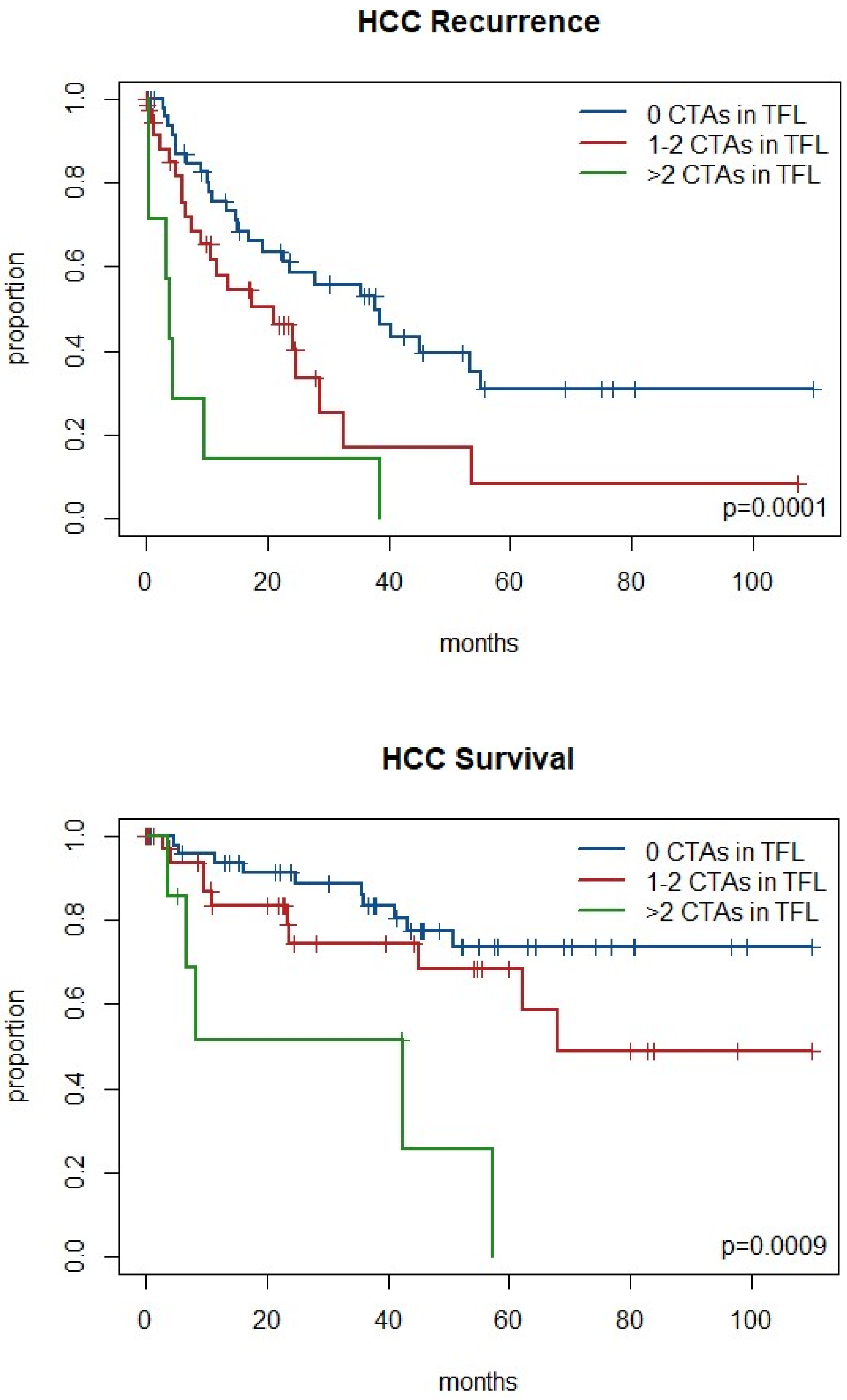


**Figure S5.** HCC recurrence and HCC-specific survival by number of CTAs expressed in the discovery cohort (based on mRNA expression) in TFL. Log-rank test.

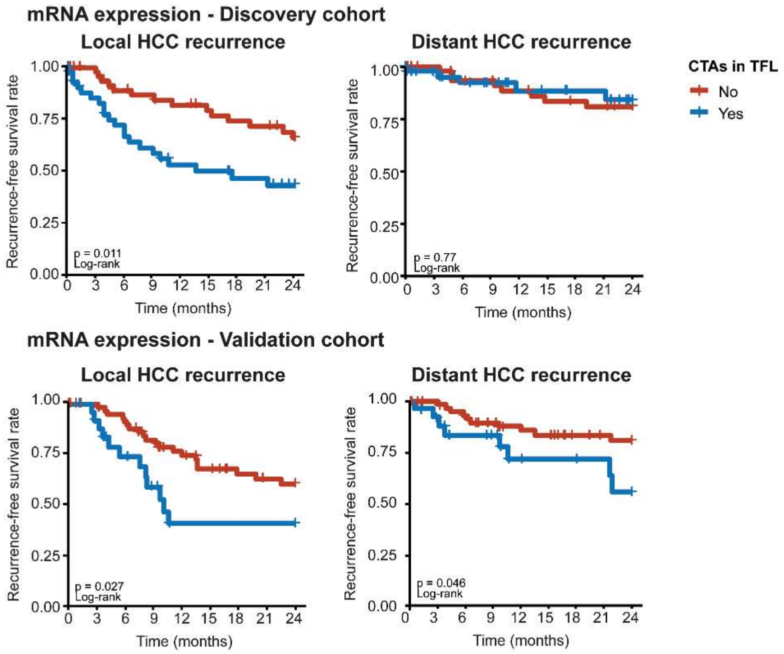


**Figure S6.** Local and distant HCC recurrence by expression of CTAs in TFL in the discovery and validation cohort. Log-rank test.

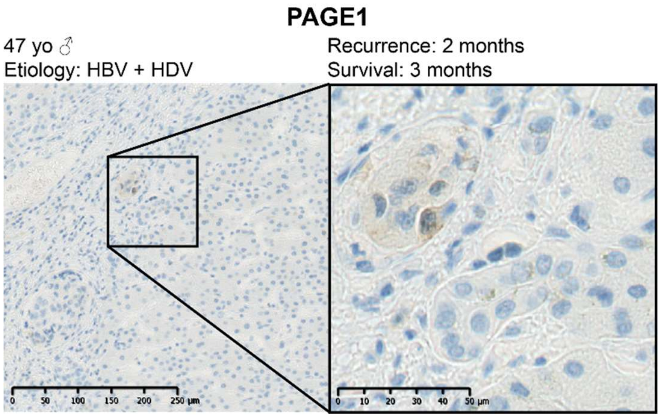


**Figure S7.** PAGE1 expressing tumor nodule in TFL. Example of IHC staining of PAGE1 protein expression in an intravascular tumor nodule in TFL, and accompanying patient data.

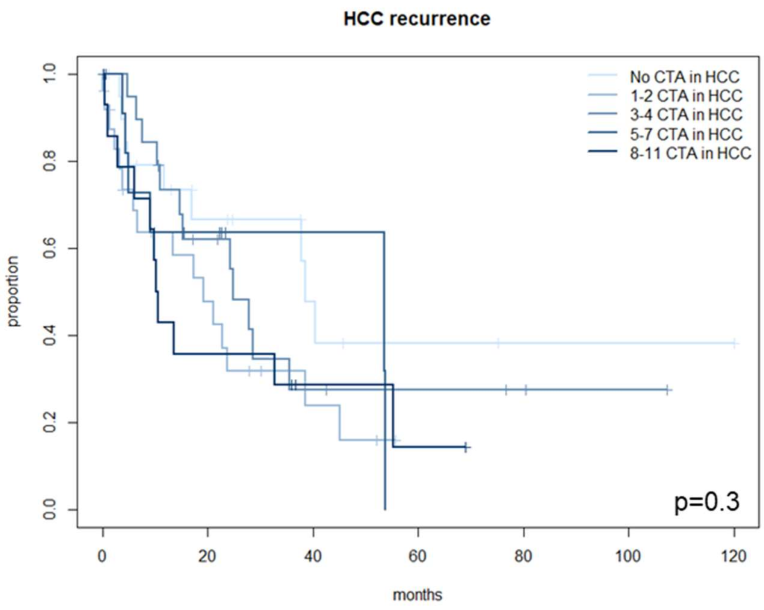


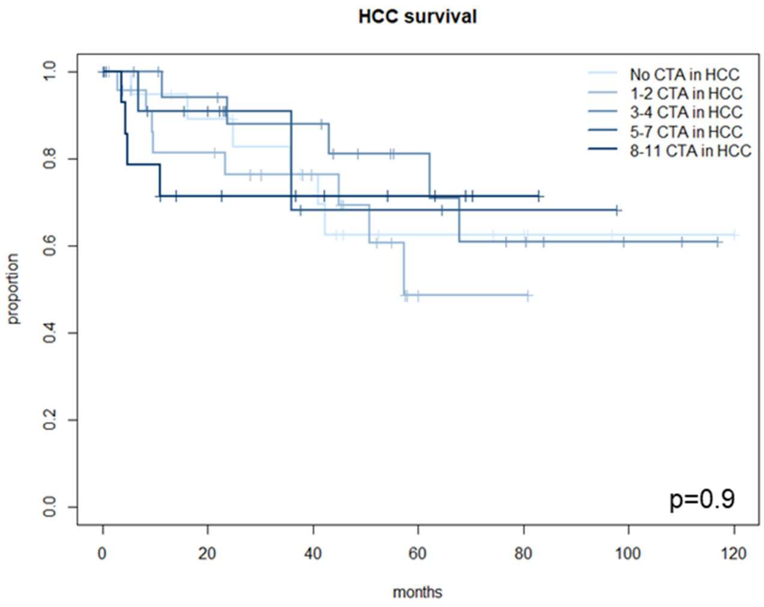


**Figure S8.** HCC recurrence and HCC-specific survival by CTA mRNA-expression in tumor tissue in the discovery cohort. Log-rank test.

## References


1. Sideras K, Biermann K, Verheij J, Takkenberg BR, Mancham S, Hansen BE, Schutz HM, de Man RA, Sprengers D, Buschow SI, et al. PD-L1, Galectin-9 and CD8(+) tumor-infiltrating lymphocytes are associated with survival in hepatocellular carcinoma. Oncoimmunology. 2017;6(2):e1273309.
2. Wei Y, Wang Y, Gong J, Rao L, Wu Z, Nie T, Shi D, Zhang L. High expression of MAGE-A9 contributes to stemness and malignancy of human hepatocellular carcinoma. Int J Oncol. 2018;52(1):219-30.
3. Jiao Y, Ding L, Chu M, Wang T, Kang J, Zhao X, Li H, Chen X, Gao Z, Gao L, et al. Effects of cancer-testis antigen, TFDP3, on cell cycle regulation and its mechanism in L-02 and HepG2 cell lines in vitro. PLoS One. 2017;12(8):e0182781.
4. Liu Q, Chen K, Liu Z, Huang Y, Zhao R, Wei L, Yu X, He J, Liu J, Qi J, et al. BORIS up-regulates OCT4 via histone methylation to promote cancer stem cell-like properties in human liver cancer cells. Cancer Lett. 2017;403:165-74.
5. Xie Y, Wang A, Lin J, Wu L, Zhang H, Yang X, Wan X, Miao R, Sang X, Zhao H. Mps1/TTK: a novel target and biomarker for cancer. J Drug Target. 2017;25(2):112-8.

6. Charoentong P, Finotello F, Angelova M, Mayer C, Efremova M, Rieder D, Hackl H, Trajanoski Z. Pan-cancer Immunogenomic Analyses Reveal Genotype-Immunophenotype Relationships and Predictors of Response to Checkpoint Blockade. Cell Rep. 2017;18(1):248-62.
7. Kido T, Lau YC. Identification of a TSPY co-expression network associated with DNA hypomethylation and tumor gene expression in somatic cancers. J Genet Genomics. 2016;43(10):577-85.
8. Fu J, Luo B, Guo WW, Zhang QM, Shi L, Hu QP, Chen F, Xiao SW, Xie XX. Down-regulation of cancer/testis antigen OY-TES-1 attenuates malignant behaviors of hepatocellular carcinoma cells in vitro. Int J Clin Exp Pathol. 2015;8(7):7786-97.
9. Wang M, Li J, Wang L, Chen X, Zhang Z, Yue D, Ping Y, Shi X, Huang L, Zhang T, et al. Combined cancer testis antigens enhanced prediction accuracy for prognosis of patients with hepatocellular carcinoma. Int J Clin Exp Pathol. 2015;8(4):3513-28.
10. Sideras K, Bots SJ, Biermann K, Sprengers D, Polak WG, JN IJ, de Man RA, Pan Q, Sleijfer S, Bruno MJ, et al. Tumour antigen expression in hepatocellular carcinoma in a low-endemic western area. Br J Cancer. 2015;112(12):1911-20.
11. Melis M, Diaz G, Kleiner DE, Zamboni F, Kabat J, Lai J, Mogavero G, Tice A, Engle RE, Becker S, et al. Viral expression and molecular profiling in liver tissue versus microdissected hepatocytes in hepatitis B virus-associated hepatocellular carcinoma. J Transl Med. 2014;12:230.
12. Li S, Mo C, Huang S, Yang S, Lu Y, Peng Q, Wang J, Deng Y, Qin X, Liu Y. Over-expressed Testis-specific Protein Y-encoded 1 as a novel biomarker for male hepatocellular carcinoma. PLoS One. 2014;9(2):e89219.
13. Deng Q, Li KY, Chen H, Dai JH, Zhai YY, Wang Q, Li N, Wang YP, Han ZG. RNA interference against cancer/testis genes identifies dual specificity phosphatase 21 as a potential therapeutic target in human hepatocellular carcinoma. Hepatology. 2014;59(2):518-30.
14. Xia QY, Liu S, Li FQ, Huang WB, Shi LN, Zhou XJ. Sperm protein 17, MAGE-C1 and NY-ESO-1 in hepatocellular carcinoma: expression frequency and their correlation with clinical parameters. Int J Clin Exp Pathol. 2013;6(8):1610-6.
15. Zhou JD, Shen F, Ji JS, Zheng K, Huang M, Wu JC. FAM9C plays an anti-apoptotic role through activation of the PI3K/Akt pathway in human hepatocellular carcinoma. Oncol Rep. 2013;30(3):1275-84.
16. Chen K, Huang W, Huang B, Wei Y, Li B, Ge Y, Qin Y. BORIS, brother of the regulator of imprinted sites, is aberrantly expressed in hepatocellular carcinoma. Genet Test Mol Biomarkers. 2013;17(2):160-5.
17. Song MH, Choi KU, Shin DH, Lee CH, Lee SY. Identification of the cancer/testis antigens AKAP3 and CTp11 by SEREX in hepatocellular carcinoma. Oncol Rep. 2012;28(5):1792-8.
18. Li H, Fang L, Xiao X, Shen L. The expression and effects the CABYR-c transcript of CABYR gene in hepatocellular carcinoma. Bull Cancer. 2012;99(3):E26-33.
19. Yoon H, Lee H, Kim HJ, You KT, Park YN, Kim H, Kim H. Tudor domain-containing protein 4 as a potential cancer/testis antigen in liver cancer. Tohoku J Exp Med. 2011;224(1):41-6.
20. Tseng YT, Hsia JY, Chen CY, Lin NT, Chong PC, Yang CY. Expression of the sperm fibrous sheath protein CABYR in human cancers and identification of $\alpha$-enolase as an interacting partner of CABYR-a. Oncol Rep. 2011;25(4):1169-75.
21. Wang XY, Chen HS, Luo S, Zhang HH, Fei R, Cai J. Comparisons for detecting NY-ESO-1 mRNA expression levels in hepatocellular carcinoma tissues. Oncol Rep. 2009;21(3):713-9.
22. Riener MO, Wild PJ, Soll C, Knuth A, Jin B, Jungbluth A, Hellerbrand C, Clavien PA, Moch H, Jochum W. Frequent expression of the novel cancer testis antigen MAGE-C2/CT-10 in hepatocellular carcinoma. Int J Cancer. 2009;124(2):352-7.
23. Lu Y, Wu LQ, Lu ZH, Wang XJ, Yang JY. Expression of SSX-1 and NY-ESO-1 mRNA in tumor tissues and its corresponding peripheral blood expression in patients with hepatocellular carcinoma. Chin Med J (Engl). 2007;120(12):1042-6.
24. Wu LQ, Lu Y, Wang XF, Lv ZH, Zhang B, Yang JY. Expression of cancer-testis antigen (CTA) in tumor tissues and peripheral blood of Chinese patients with hepatocellular carcinoma. Life Sci. 2006;79(8):744-8.
25. Watanabe T, Suda T, Tsunoda T, Uchida N, Ura K, Kato T, Hasegawa S, Satoh S, Ohgi S, Tahara H, et al. Identification of immunoglobulin superfamily 11 (IGSF11) as a novel target for cancer immunotherapy of gastrointestinal and hepatocellular carcinomas. Cancer Sci. 2005;96(8):498-506.
26. Yin YH, Li YY, Qiao H, Wang HC, Yang XA, Zhang HG, Pang XW, Zhang Y, Chen WF. TSPY is a cancer testis antigen expressed in human hepatocellular carcinoma. Br J Cancer. 2005;93(4):458-63.
27. Shi YY, Wang HC, Yin YH, Sun WS, Li Y, Zhang CQ, Wang Y, Wang S, Chen WF. Identification and analysis of tumour-associated antigens in hepatocellular carcinoma. Br J Cancer. 2005;92(5):929-34.
28. Peng JR, Chen HS, Mou DC, Cao J, Cong X, Qin LL, Wei L, Leng XS, Wang Y, Chen WF. Expression of cancer/testis (CT) antigens in Chinese hepatocellular carcinoma and its correlation with clinical parameters. Cancer Lett. 2005;219(2):223-32.
29. Sato S, Noguchi Y, Wada H, Fujita S, Nakamura S, Tanaka R, Nakada T, Hasegawa K, Nakagawa K, Koizumi F, et al. Quantitative real-time RT-PCR analysis of NY-ESO-1 and LAGE-1a mRNA expression in normal tissues and tumors, and correlation of the protein expression with the mRNA copy number. Int J Oncol. 2005;26(1):57-63.
30. Yang XA, Dong XY, Qiao H, Wang YD, Peng JR, Li Y, Pang XW, Tian C, Chen WF. Immunohistochemical analysis of the expression of FATE/BJ-HCC-2 antigen in normal and malignant tissues. Lab Invest. 2005;85(2):205-13.
31. Dong XY, Li YY, Yang XA, Chen WF. BJ-HCC-20, a potential novel cancer-testis antigen. Biochem Cell Biol. 2004;82(5):577-82.
32. Dong XY, Yang XA, Wang YD, Chen WF. Zinc-finger protein ZNF165 is a novel cancer-testis antigen capable of eliciting antibody response in hepatocellular carcinoma patients. Br J Cancer. 2004;91(8):1566-70.
33. Zhao L, Mou DC, Leng XS, Peng JR, Wang WX, Huang L, Li S, Zhu JY. Expression of cancer-testis antigens in hepatocellular carcinoma. World J Gastroenterol. 2004;10(14):2034-8.
34. Li B, Qian XP, Pang XW, Zou WZ, Wang YP, Wu HY, Chen WF. HCA587 antigen expression in normal tissues and cancers: correlation with tumor differentiation in hepatocellular carcinoma. Lab Invest. 2003;83(8):1185-92.

35. Dong XY, Su YR, Qian XP, Yang XA, Pang XW, Wu HY, Chen WF. Identification of two novel CT antigens and their capacity to elicit antibody response in hepatocellular carcinoma patients. Br J Cancer. 2003;89(2):291-7.
36. Luo G, Huang S, Xie X, Stockert E, Chen YT, Kubuschok B, Pfreundschuh M. Expression of cancer-testis genes in human hepatocellular carcinomas. Cancer Immun. 2002;2:11.
37. Wang Y, Han KJ, Pang XW, Vaughan HA, Qu W, Dong XY, Peng JR, Zhao HT, Rui JA, Leng XS, et al. Large scale identification of human hepatocellular carcinoma-associated antigens by autoantibodies. J Immunol. 2002;169(2):1102-9.
38. de Wit NJ, Weidle UH, Ruiter DJ, van Muijen GN. Expression profiling of MMA-1a and splice variant MMA-1b: new cancer/testis antigens identified in human melanoma. Int J Cancer. 2002;98(4):547-53.
39. Ono T, Kurashige T, Harada N, Noguchi Y, Saika T, Niikawa N, Aoe M, Nakamura S, Higashi T, Hiraki A, et al. Identification of proacrosin binding protein sp32 precursor as a human cancer/testis antigen. Proc Natl Acad Sci U S A. 2001;98(6):3282-7.
40. Chen CH, Chen GJ, Lee HS, Huang GT, Yang PM, Tsai LJ, Chen DS, Sheu JC. Expressions of cancer-testis antigens in human hepatocellular carcinomas. Cancer Lett. 2001;164(2):189-95.
41. Bergeron A, Picard V, LaRue H, Harel F, Hovington H, Lacombe L, Fradet Y. High frequency of MAGE-A4 and MAGE-A9 expression in high-risk bladder cancer. Int J Cancer. 2009;125(6):1365-71.
42. Schnieders F, Dork T, Arnemann J, Vogel T, Werner M, Schmidtke J. Testis-specific protein, Y-encoded (TSPY) expression in testicular tissues. Hum Mol Genet. 1996;5(11):1801-7.
